# The Survival Effect of Radiotherapy on Stage II/III Rectal Cancer in Different Age Groups: Formulating Radiotherapy Decision-Making Based on Age

**DOI:** 10.3389/fonc.2021.695640

**Published:** 2021-07-28

**Authors:** Yuqiang Li, Heli Liu, Yuan Zhou, Zhongyi Zhou, Wenxue Liu, Lilan Zhao, Cenap Güngör, Dan Wang, Qian Pei, Haiping Pei, Fengbo Tan

**Affiliations:** ^1^Department of General Surgery, Xiangya Hospital, Central South University, Changsha, China; ^2^Department of General Visceral and Thoracic Surgery, University Medical Center Hamburg-Eppendorf, Hamburg, Germany; ^3^Department of Cardiology, Xiangya Hospital, Central South University, Changsha, China; ^4^Department of Thoracic Surgery, Fujian Provincial Hospital, Fuzhou, China

**Keywords:** radiotherapy, age, overall survival, SEER (the Surveillance, Epidemiology, and End Results) database, rectal cancer

## Abstract

**Introduction:**

Total mesorectal excision (TME), chemotherapy (CT), and radiotherapy (RT) are usually integrated into the comprehensive treatment of stage II/III rectal cancer (RC). Neoadjuvant radiotherapy (nRT) has become the standard treatment for stage II/III RC patients to help reduce the size of a tumor or kill cancer cells that have spread. Adjuvant RT is delivered after the resection to destroy remaining cancer cells and used mainly in stage II/III RC patients who have not received preoperative radiotherapy, such as those who suffered from a bowel obstruction before surgery. It is controversial whether radiotherapy can improve the survival of stage II/III RC patients. An increasing number of studies have reported that rectal cancer exhibited mismatched biology, epidemiology, and therapeutic response to current treatment strategy in different age groups. It is necessary to investigate whether radiotherapy exhibits disparate effects in different age groups of patients with stage II/III RC.

**Methods:**

Data from the Surveillance, Epidemiology, and End Results (SEER) Program was extracted to identify stage II/III RC diagnosed in the periods of 2004–2016. The statistical methods included Pearson’s chi-square test, log-rank test, Cox regression model, and propensity score matching.

**Results:**

Neoadjuvant radiotherapy (nRT) cannot improve the prognosis, and postoperative RT may even reduce the survival time for early onset stage II/III RC. Postoperative RT was not able to improve the overall survival (OS), while nRT may provide limited survival improvement for middle-aged stage II/III RC patients. In addition, radiotherapy can significantly improve the prognosis for elderly stage II/III RC.

**Conclusions:**

This study indicated the inconsistent survival effect of radiotherapy on stage II/III rectal cancer patients in different age groups. Hence, we formulated a novel flow chart of radiotherapy decision-making based on age in stage II/III RC patients.

## Introduction

Colorectal cancer (CRC) is ranked in the top third malignancy in males and the second in females (1) and includes approximately 30–50% rectal cancer (RC) (2). Total mesorectal excision (TME), chemotherapy (CT) and radiotherapy (RT) are usually integrated into the comprehensive treatment of stage II/III RC.

RT, which directly delivers ionizing radiation to the target area including the primary tumor and regional lymph nodes, may cause genetic damage, such as irradiation-induced DNA double-strand breaks, and can ultimately lead to apoptosis (3). Neoadjuvant radiotherapy (nRT) has become the standard treatment for stage II/III RC patients to help reduce the size of a tumor or kill cancer cells that have spread. Adjuvant RT is delivered after the resection to destroy remaining cancer cells and used mainly in stage II/III RC patients who have not received preoperative radiotherapy, such as those who suffered from a bowel obstruction before surgery. Prior clinical studies demonstrated that nRT could provide benefits to solid malignancies by inducing tumor downstaging and reducing local recurrence (4–6). However, it is controversial whether radiotherapy, including nRT and adjuvant RT, can improve the survival of stage II/III RC patients. A meta-analysis indicated that the survival benefits from radiotherapy failed to reach statistical significance in patients with rectal cancer (7). Nonetheless, another clinical research study illustrated that radiotherapy was able to prolong survival of rectal cancer patients (8).

An increasing number of studies have reported that rectal cancer exhibited mismatched biology, epidemiology, and therapeutic response to current treatment strategy in different age groups (9). Early onset rectal cancer patients are correlated with more unfavorable phenotypes and more aggressive biological behaviors (10). Meanwhile, as the incidence of rectal cancer in elderly patients has decreased, an inverse trend has been monitored in adults younger than age 50 (early onset RC) (11). In addition, younger RC patients tend to present with advanced disease, with more than 60% of those with early onset RC diagnosed with lymph nodes and even distant metastasis (12). Furthermore, a previous study found that young individuals failed to obtain equivalent survival benefits from chemotherapy compared to elderly patients with colorectal cancer (13). It is necessary to investigate whether radiotherapy exhibits disparate effects in different age groups of patients with stage II/III RC.

To address this gap in our knowledge and to verify the hypothesis that the survival effect of radiotherapy in stage II/III RC patients may be inconsistent among different age groups, we aimed to analyze overall survival by dividing stage II/III RC into early onset, middle-aged, and elderly cohorts. This work queried a large national database, specifically the Surveillance, Epidemiology, and End Results (SEER) linked database, for patients with rectal cancer who underwent proctectomy treatment to compare outcomes in cohorts defined by adherence to radiotherapy and age.

## Materials And Methods

### Patient Screening

**Figure 1:**
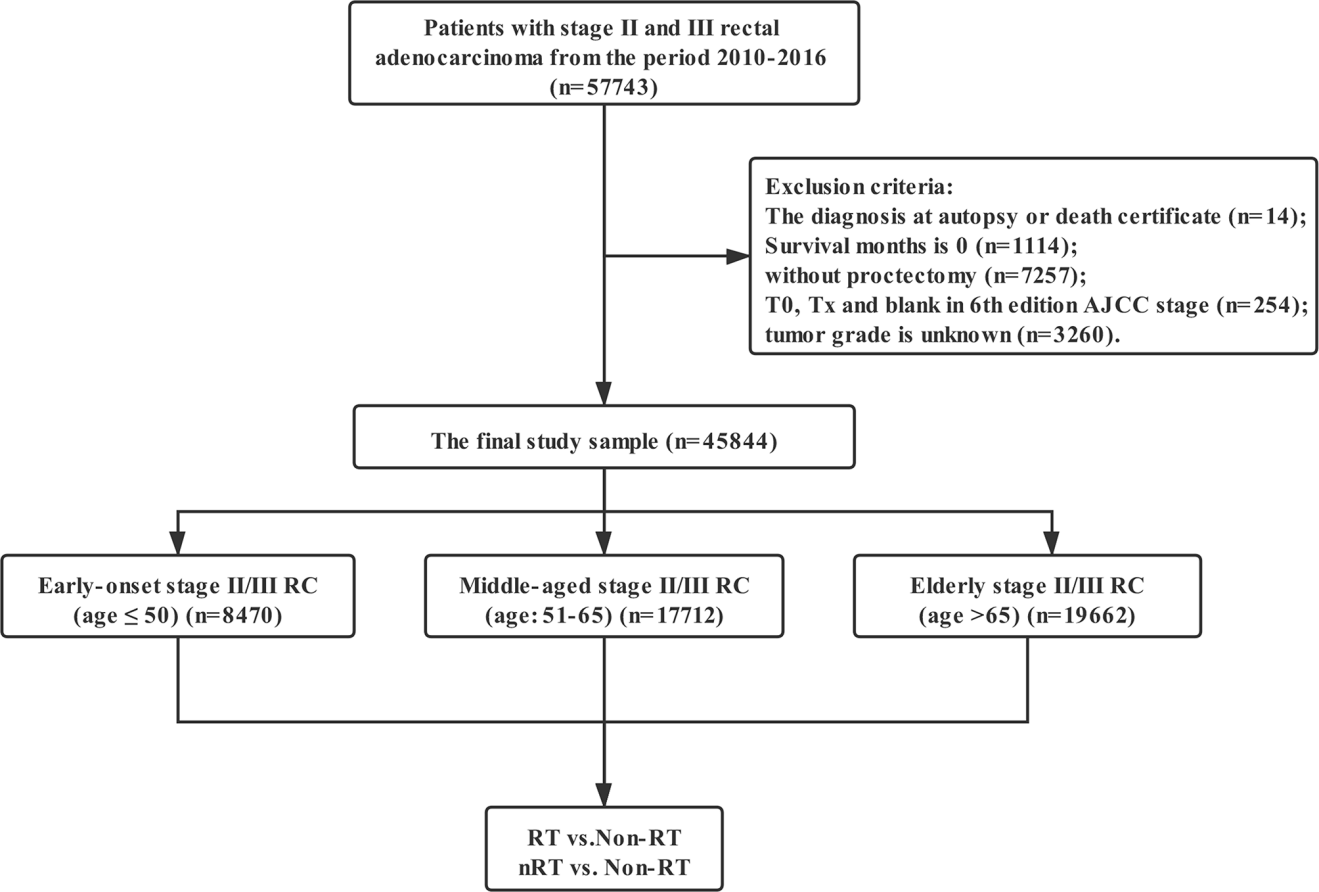
The flow diagram.

Data in this retrospective analysis were extracted from the SEER database, which collects data on cancer cases from various locations and sources throughout the United States. SEER is supported by the Surveillance Research Program (SRP) in NCI’s Division of Cancer Control and Population Sciences (DCCPS). The target population was limited to patients with stage II and III rectal adenocarcinoma (ICD-O-3: 8140, 8141, 8144, 8201, 8210, 8211, 8213, 8245, 8255, 8260, 8261, 8262, 8263, 8310, 8323, 8480, 8481, 8490) diagnosed in the periods of 2004–2016, 57,743 patients in total. Exclusion criteria: the diagnosis at autopsy or death certificate (n = 14); Survival months is 0 (n = 1,114); without proctectomy (n = 7,257); T0, Tx, and blank in 6th edition AJCC stage (n = 254); tumor grade is unknown (n = 3,260). The final study sample contained 45,844 patients (**Figure 1**).

### Statistical Analysis

Intergroup comparisons were analyzed using Pearson’s chi-square test. Log-rank test was used to compare overall survival (OS) between different groups. A hazard ratio (HR) and a 95% confidence interval (CI) were evaluated by a multivariate Cox proportional hazards regression model. All variables were included directly in the Cox regression model for multivariate analysis. In order to eliminate the influence of other variables, we conducted a propensity score matching (PSM). Statistical analyses were performed with IBM SPSS statistics trial ver. 25.0 (IBM, Armonk, NY, USA). All reported p-values lower than 0.05 were considered significant.

## Results

### Patients Characteristics

The characteristics of 45,844 patients with locally advanced rectal cancer enrolled from the SEER database are summarized in **Table 1**. The total population included 8,470 patients with early onset stage II/III RC (age ≤ 50), 17,712 cases of middle-aged stage II/III RC (age: 51–65), and 19,662 elderly patients with stage II/III RC (age > 65). The three groups exhibited significant differences regarding clinicopathological factors. The ratio of T3–4 in the elderly stage II/III RC patients was significantly higher than that in the other two cohorts (p < 0.001). The proportion of stage II/III RC patients with metastatic lymph nodes was the highest among the early onset group (5,658, 66.80%), followed by middle-aged (10,708, 60.46%) and older adults (10,435, 53.07%) (p < 0.001). Moreover, stage II/III RC treatment decision-making and execution seem to be affected by age. Elderly stage II/III RC patients tend to give up chemotherapy (non-CT: 44.12%) and radiotherapy (non-RT: 53.38%) as well as received proctectomy with RNE <12 (34.97%) compared to early onset (non-CT: 12.66%; non-RT: 28.85%; RNE < 12: 24.88%), and middle-aged patients (non-CT: 19.00%; non-RT: 33.17%; RNE < 12: 30.49%).

**Table 1:** Characteristics of stage II/III rectal cancer.

Characteristics	Total (n = 45,844)		Early onset stage II/III RC (n = 8,470)		Middle-aged stage II/III RC (n = 17,712)		Elderly stage II/III RC (n = 19,662)		*p*-value
	N	%	N	%	N	%	N	%	
Gender									0.004
Female	18,905	41.2%	3,685	43.5%	6,691	37.8%	8529	43.4%	
Male	26,939	58.8%	4,785	56.5%	11,021	62.2%	11133	56.6%	
Marital status									<0.001
Married	26,541	57.9%	5,175	61.1%	10,838	61.2%	10528	53.5%	
Unmarried/NOS	19,303	42.1%	3,295	38.9%	6,874	38.8%	9134	46.5%	
Race									<0.001
White	37,052	80.8%	6,668	78.7%	14,095	79.6%	16289	82.8%	
Non-white	8,792	19.2%	1,802	21.3%	3,617	20.4%	3373	17.2%	
Pathologic grade									0.816
Grade I/II	38,411	83.8%	7,006	82.7%	15,008	84.7%	16397	83.4%	
Grade III/IV	7,433	16.2%	1,464	17.3%	2,704	15.3%	3265	16.6%	
Histologic type									0.110
Adenocarcinomas	42,518	92.7%	7,839	92.6%	16,526	93.3%	18153	92.3%	
MCC/SRCC	3,326	7.3%	631	7.4%	1,186	6.7%	1509	7.7%	
T staging									<0.001
T1–2	4,874	10.6%	985	11.6%	2,026	11.4%	1863	9.5%	
T3–4	40,970	89.4%	7,485	88.4%	15,686	88.6%	17799	90.5%	
N staging									<0.001
N0	19,043	41.5%	2,812	33.2%	7,004	39.5%	9227	46.9%	
N+	26,801	58.5%	5,658	66.8%	10,708	60.5%	10435	53.1%	
Radiotherapy									<0.001
Non-RT	18,814	41.0%	2,444	28.8%	5,875	33.2%	10495	53.4%	
RT	8,149	17.8%	1,626	19.2%	3,510	19.8%	3013	15.3%	
nRT	18,881	41.2%	4,400	52.0%	8,327	47.0%	6154	31.3%	
Chemotherapy									<0.001
No	13,112	28.6%	1,072	12.7%	3,366	19.0%	8674	44.1%	
Yes	32,732	71.4%	7,398	87.3%	14,346	81.0%	10988	55.9%	
RNE									<0.001
<12	14,382	31.4%	2,107	24.9%	5,400	30.5%	6875	35.0%	
≥12	31,145	67.9%	6,294	74.3%	12,200	68.9%	12651	64.3%	
NOS	317	0.7%	69	0.8%	112	0.6%	136	0.7%	
CEA									<0.001
Negative	16,429	35.8%	3,389	40.0%	6,594	37.2%	6446	32.8%	
Positive	12,413	27.1%	2,205	26.0%	4,914	27.8%	5294	26.9%	
NOS	17,002	37.1%	2,876	34.0%	6,204	35.0%	7922	40.3%	
Tumor size (cm)									<0.001
≤5cm	27,656	60.3%	4,858	57.4%	10,610	59.9%	12188	62.0%	
>5cm	13,287	29.0%	2,562	30.2%	5,013	28.3%	5712	29.0%	
NOS	4,901	10.7%	1,050	12.4%	2,089	11.8%	1762	9.0%	

MCC, mucinous cell carcinoma; SRCC, signet ring cell carcinoma; RNE, regional nodes examined; nRT: neoradiotherapy; RT, radiotherapy (not neoadjuvant); NOS, not otherwise specified.

### The Effect of Radiotherapy on Early Onset Stage II/III RC

**Figure 2:**
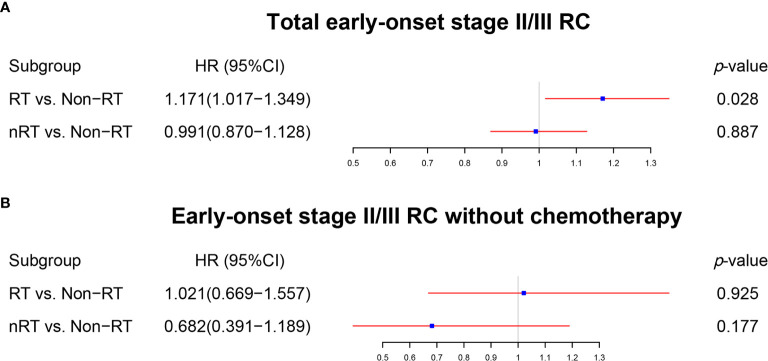
The forest plot was used to show the results of the multivariable Cox regression in early onset stage II/III RC. **(A)** RT *vs*. non-RT and nRT *vs*. non-RT in total early onset stage II/III RC patients. **(B)** RT *vs*. non-RT and nRT *vs*. non-RT in early onset stage II/III RC patients without chemotherapy. (The results were extracted from **Table S1**).

**Figure 3:**
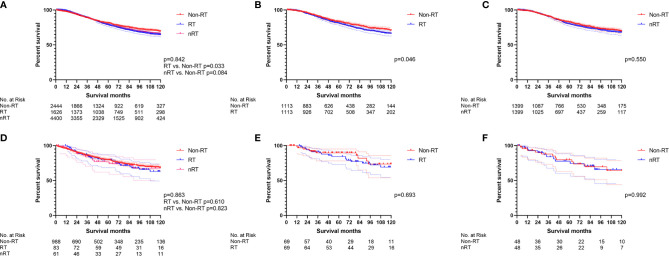
The survival curves were used to demonstrate the effect of radiotherapy in early onset stage II/III RC patients. **(A)** The total early onset stage II/III RC patients before PSM. **(B)** RT *vs*. non-RT in all early onset stage II/III RC patients after PSM. **(C)** nRT *vs*. non-RT in all early onset stage II/III RC patients after PSM. **(D)** The early onset stage II/III RC patients without chemotherapy before PSM. **(E)** RT *vs*. non-RT in early onset stage II/III RC patients without chemotherapy after PSM. **(F) n**RT *vs*. non-RT in early onset stage II/III RC patients without chemotherapy after PSM. (The results of PSM are summarized in **Table S2**).

The survival effect of radiotherapy on early onset stage II/III RC was analyzed first. The multivariate Cox regression models (**Figure 2A**, **Table S1**) displayed that nRT was not able to improve OS (p = 0.887), while RT played a risk factor (p = 0.028, HR = 1.171) in early onset stage II/III RC patients. Similar results were obtained by the survival analysis before PSM (**Figure 3A**; RT *vs*. non-RT: p = 0.033; nRT *vs*. non-RT: p = 0.084). PSM was then used to eliminate the influence of other variables (**Table S2**). Unfortunately, early onset stage II/III RC patients with radiotherapy developed worse OS compared to those without radiotherapy (**Figure 3B**, p = 0.046). In addition, there was no significant survival difference between nRT and non-RT (**Figure 3C**, p = 0.550). Afterward, for further analysis, patients without chemotherapy were extracted to eliminate the impact of chemotherapy used. Both the multivariate Cox regression analysis (**Figure 2B**, **Table S1**) and the survival analysis before PSM (**Figure 3D**) indicated that radiotherapy, including nRT and RT, cannot significantly affect the OS of early onset stage II/III RC patients. The survival curves after PSM (**Table S2**) confirmed the previous results (**Figure 3E**: RT *vs*. non-RT, p = 0.693; **Figure 3F**: nRT *vs*. non-RT, p = 0.992). Collectively, nRT cannot improve the prognosis, and RT may even reduce the survival time for early onset stage II/III RC.

### The Effect of Radiotherapy on Middle-Aged Patient With Stage II/III RC

**Figure 4:**
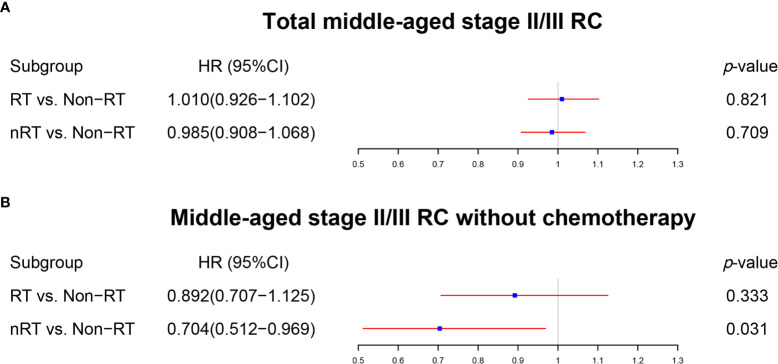
The forest plot was used to show the results of the multivariable Cox regression in middle-aged stage II/III RC patients. **(A)** RT *vs*. non-RT and nRT *vs*. non-RT in total middle-aged stage II/III RC patients. **(B)** RT *vs*. non-RT and nRT *vs*. non-RT in middle-aged stage II/III RC patients without chemotherapy. (The results were extracted from **Table S3**).

**Figure 5:**
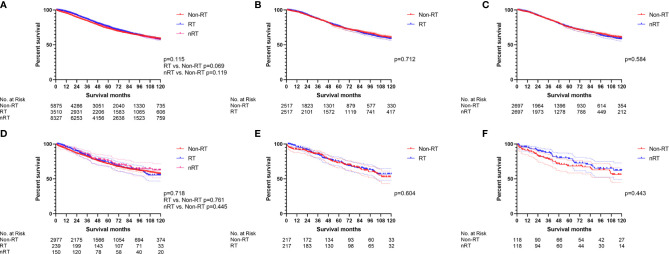
The survival curves were applied to display the effect of radiotherapy in middle-aged stage II/III RC patients. All survival comparisons failed to reach statistical differences. **(A)** The total middle-aged stage II/III RC patients before PSM. **(B)** RT *vs*. non-RT in all middle-aged stage II/III RC patients after PSM. **(C)** nRT *vs*. non-RT in all middle-aged stage II/III RC patients after PSM. **(D)** Middle-aged stage II/III RC patients without chemotherapy before PSM. **(E)** RT *vs*. non-RT in middle-aged stage II/III RC patients without chemotherapy after PSM. **(F)** nRT *vs*. non-RT in middle-aged stage II/III RC patients without chemotherapy after PSM. (The results of PSM are summarized in **Table S4**).

The same methods were applied to analyze middle-aged patients with stage II/III RC. The multivariate Cox regression analysis (**Figure 4A**, **Table S3**) and the survival analysis before PSM (**Figure 5A**) illustrated that both RT and nRT failed to prolong OS of middle-aged patients with stage II/III RC, which was further confirmed by survival analysis after PSM (**Table S4** **;** **Figure 5B**: RT *vs*. non-RT, p = 0.712; **Figure 5C**: nRT *vs*. non-RT, p = 0.584). Interestingly, nRT can be used as a prognostic factor in the multivariate Cox regression model analyzing middle-aged stage II/III RC patients without chemotherapy (**Figure 4B**, **Table S3**). However, the survival curves did not show the survival advantage of radiotherapy for middle-aged stage II/III RC patients (before PSM: **Figure 5D**, p = 0.718; RT *vs*. non-RT, p = 0.761; nRT *vs*. non-RT, p = 0.445) (after PSM: **Table S4** **;** **Figure 5E**, RT *vs*. non-RT, p = 0.604; **Figure 5F**, nRT *vs*. non-RT, p = 0.443). Aggregately, RT was not able to improve the OS, while nRT may provide limited survival improvement for middle-aged stage II/III RC patients.

### The Effect of Radiotherapy on Elderly Stage II/III RC Patients

**Figure 6:**
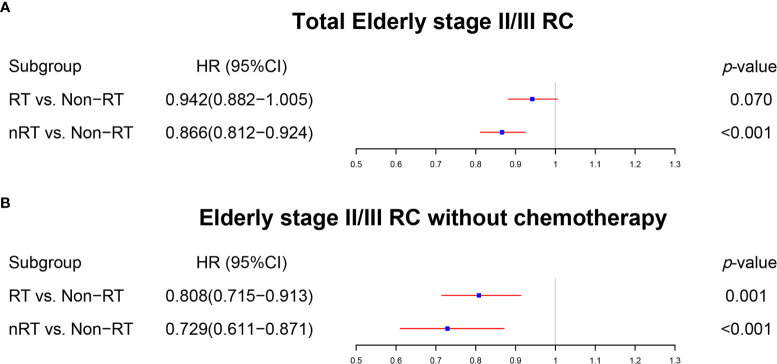
The forest plot was used to show the results of the multivariable Cox regression in elderly stage II/III RC. **(A)** RT *vs*. non-RT and nRT *vs*. non-RT in total elderly stage II/III RC patients. **(B)** RT *vs*. non-RT and nRT *vs*. non-RT in elderly stage II/III RC patients without chemotherapy. (The results were extracted from **Table S5**).

**Figure 7:**
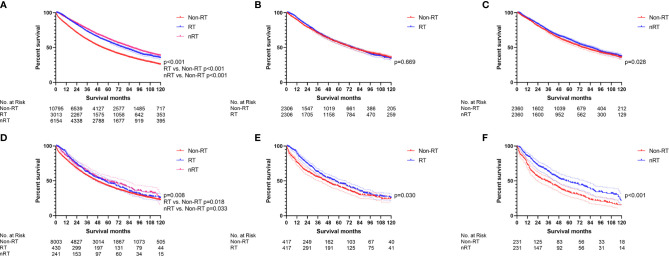
The survival curves were utilized to indicate the effect of radiotherapy in elderly stage II/III RC patients. **(A)** The total elderly stage II/III RC patients before PSM. **(B)** RT *vs*. non-RT in all elderly stage II/III RC patients after PSM. **(C)** nRT *vs*. non-RT in all elderly stage II/III RC patients after PSM. **(D)** The total elderly stage II/III RC patients without chemotherapy before PSM. **(E)** RT *vs*. non-RT in elderly stage II/III RC patients without chemotherapy after PSM. **(F)** nRT *vs*. non-RT in elderly stage II/III RC patients without chemotherapy after PSM. (The results of PSM are summarized in **Table S6**).

Radiotherapy played a key role in the treatment of over-65 stage II/III RC patients. Although they failed to obtain significant benefit from RT (p = 0.070), over-65 stage II/III RC patients received superior survival from nRT (p < 0.001) in the multivariate Cox regression model (**Figure 6A**, **Table S5**). The survival curves without PSM demonstrated that both RT and nRT provided survival benefit to elderly stage II/III RC patients (**Figure 7A**, p < 0.001; RT *vs*. non-RT, p < 0.001; nRT *vs*. non-RT, p < 0.001). However, the survival curves with PSM (**Table S6**) showed indistinguishable survival between RT and non-RT groups in elderly stage II/III RC patients (**Figure 7B**, p = 0.669). Nevertheless, nRT can still provide significant survival benefits to elderly patients with stage II/III RC after PSM (**Figure 7C**, p = 0.028). Among the elderly stage II/III RC patients, 8,647 who did not receive chemotherapy were used for further analysis. Intriguingly, both of the multivariate Cox regression analysis (**Figure 6B**, **Table S5**) and the survival analysis before PSM (**Figure 7D**) indicated that radiotherapy, including nRT and RT, can prolong OS of elderly stage II/III RC patients, which was verified by the survival curves using data after PSM (**Table S6**) (**Figure 7E**: RT *vs*. non-RT, p = 0.030; **Figure 7F**: nRT *vs*. non-RT, p < 0.001). To sum up, radiotherapy can significantly improve the prognosis of over-65 stage II/III RC patients.

## Discussion

This study focused on a possible important causality between radiotherapy and the age of stage II/III RC patients. To the best of our knowledge, this study was the first research to specifically investigate the survival effect of radiotherapy on different ages of patients with stage II/III RC. Numerous tumors exhibit differences in molecular background, biological behavior, etiology as well as therapeutic response among various age groups (9, 14–18). A recent research study focusing on young breast cancer patients found that chemotherapy was an insignificant prognostic factor in the multivariable analysis with Cox regression for overall survival and cancer-specific survival (19), which is obviously inconsistent with most studies without age grouping. Moreover, young individuals failed to obtain equivalent survival benefits from chemotherapy compared to over-65 patients with colorectal cancer (13). This evidence drove us to explore the differentiated impact of radiotherapy in various age groups of stage II/III RC patients. The results of this study using a large national database indicated the inconsistent survival effect of radiotherapy on stage II/III rectal cancer patients in different age groups, which imply that age should be used as a deciding factor for radiotherapy for stage II/III RC patients.

Use of radiotherapy in the treatment of stage II/III RC patients continues to evolve (20). The sphincter preservation is the main issue affecting the quality of life faced by rectal cancer patients. Therefore, we planned to discuss radiotherapy-decision-making according to survival prolongation combined with sphincter preservation for patients with stage II/III RC. The CAO/ARO/AIO-94 trial indicated that neoadjuvant chemoradiotherapy (nCRT) was associated with a significant reduction in local recurrence and treatment-associated toxicity compared to postoperative chemoradiotherapy (CRT) (5, 21). Meanwhile, preoperative chemoradiotherapy demonstrated increased rates of pathological complete response (pCR) and improved local disease recurrence rates relative to chemotherapy (22) or radiotherapy alone (6, 23, 24). Hence, neoadjuvant chemoradiotherapy (nCRT) is still the first choice for those mid-low rectal cancer patients with influence on the sphincter preservation. In fact, radiotherapy cannot play the best role without chemotherapy, which is considered as a sensitizer for radiotherapy (25). The results from patients who received radiotherapy without chemotherapy as an unconventional treatment can only be considered as secondary evidence. Therefore, the multivariate Cox regression model analyzing middle-aged stage II/III RC patients without chemotherapy could be an inferior evidence to suggest that nRT can be used as an alternative option for middle-aged stage II/III RC patients who cannot tolerate CT.

Phase III FOWARC trial demonstrated a non-significant difference in survival outcomes between neoadjuvant chemotherapy (nCT) and nCRT (26). In fact, nCT alone, which is able to spare patients the morbidities associated with radiation, should herein be recommended as a first-line treatment for stage II/III RC patients without influencing the sphincter preservation, which is also supported by the National Comprehensive Cancer Network (NCCN) Clinical Practice Guidelines (20). Nevertheless, postoperative radiotherapy should be avoided for early onset and middle-aged stage II/III RC patients, regardless if they received neoadjuvant treatment or not, due to the ineffective or even harmful effects of RT for them.

**Figure 8:**
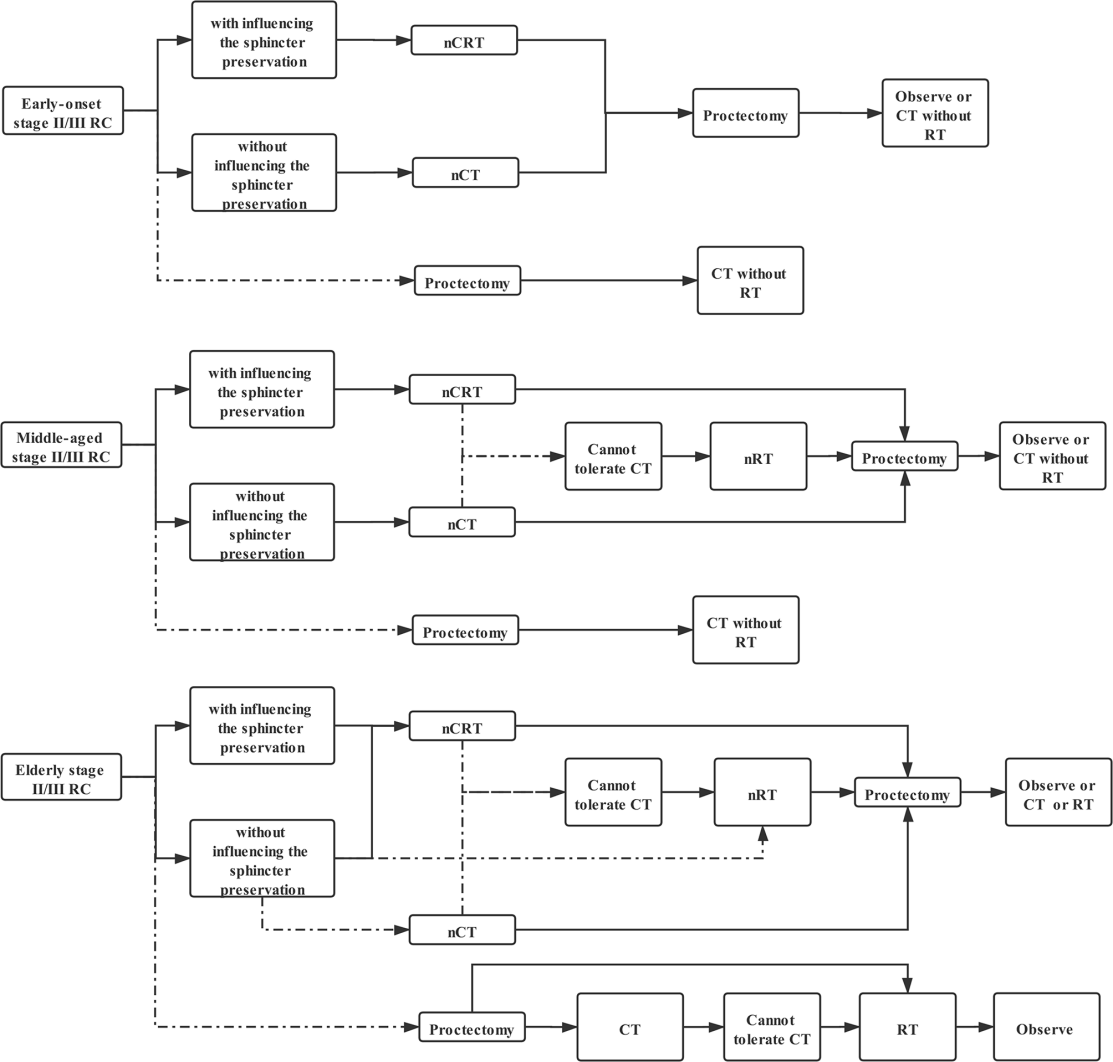
The flow chart of radiotherapy decision-making based on age in stage II/III RC patients. (Solid line: preferred option; dotted line: alternative choice).

Clinicians should pay more attention to the application of radiotherapy in over-65 stage II/III RC patients. In fact, the results of the Phase III FOWARC trial, which excluded patients older than 75, cannot be fully applied to elderly stage II/III RC (26). It is necessary to update radiotherapy strategies suitable for elderly individuals. First of all, the evidence that nRT significantly improved the prognosis of elderly stage II/III RC in the analysis including patients with chemotherapy supported that nCRT should take precedence over nCT in all elderly stage II/III RC patients. In addition, the analysis excluding patients with chemotherapy certified that nRT alone was able to prolong OS of elderly individuals with stage II/III RC, which can be used as reliable evidence to sustain that nRT was an alternative option for those who cannot tolerate chemotherapy as well as a preferred choice for some frail elderly patients without influencing the sphincter preservation. Moreover, postoperative radiotherapy can only be recommended to elderly stage II/III RC patients. However, elderly stage II/III RC patients should cautiously receive postoperative combined radiotherapy and chemotherapy, which may be too aggressive for older adults, since RT failed to provide survival benefit compared with non-RT in the analysis including those patients with chemotherapy, while RT was able to improve OS when the analysis excluded elderly patients with chemotherapy. Collectively, we can summarize a novel flow chart of radiotherapy decision-making based on age (**Figure 8**).

Our research also displayed, to a certain extent, that nRT was superior to postoperative RT. For instance, RT reduced the OS of patients with early onset stage II/III RC, while nRT did not; there was a significant survival difference between nRT and non-RT in the multivariate Cox regression model analyzing middle-aged stage II/III RC patients without chemotherapy; nRT provided more obvious survival benefits to elderly stage II/III RC patients compared to RT. Putative advantages of nRT, as opposed to RT given postoperatively, are related to both tumor volume and preservation of normal tissue (21, 27, 28). Irradiating tissue that is surgery-naïve and thus better oxygenated may result in increased sensitivity to RT. Furthermore, nRT can avoid the occurrence of radiation-induced injury to small bowel trapped in the pelvis by post-surgical adhesion. nRT that includes structures that will increase the likelihood that an anastomosis with healthy colon can be performed.

Mismatched biology may be responsible for the inconsistent survival benefits among different age groups. A recent research study reported that young rectal cancer patients have a higher proportion of cancer stem cells (CSCs) (29), which is considered as an important factor reflecting radiotherapy resistance in rectal cancer (30, 31). In addition, we took out stage II RC patients separately for analysis and got similar results comparing to the total stage II/III RC patients (**Supplementary Files**). In fact, stage II/III RC is often discussed as a whole because of consistent treatment strategies. Therefore, it is reasonable for stage II/III RC to be studied as a whole. Moreover, the results displayed that there was more RNE ≥12 in the early onset group, which implied that young patients were more likely to receive extensive treatment. Therefore, we conducted further analysis and found that these elderly stage II/III RC patients with RNE ≥12 can get survival benefit from nRT but not postoperative RT, which is similar to the results of the total elderly stage II/III RC patients, while early onset and middle-aged ones with RNE <12 cannot obtain survival benefit from radiotherapy, including nRT and postoperative RT (**Supplementary Files**). Overall, it is reliable that age is an important factor affecting the efficacy of radiotherapy in stage II/III RC patients.

However, the evidence effect of our study is weakened by some limitations. Firstly, despite that the PSM method was performed to reduce the confounding factors of independent features, some biases were inevitable due to the retrospective nature of this study. Secondarily, detailed information regarding chemotherapy and surgery for included patients was not recorded in the SEER database, which, to some extent, weakened the evidence effect of this study because young patients were more likely to receive multimodal treatment compared to the middle-aged and elderly RC patients. We used RNE as the priority for the assessment of the quality of surgery, which was mentioned in the previous studies (32, 33), since the SEER database failed to provide information regarding TME as well as surgical R-stage, which may be serious confounding factors with regard to the other modalities. Undoubtedly, the lack of data regarding surgical R-stage and the distance from the primary tumor to the mesorectal fascia weakened the reliability of the conclusions in this study. Furthermore, the SEER database does not provide detailed information about the distance between the tumor and the anus, which is one of the decisive factors that affect the decision-making of radiotherapy for RC. A recent research showed that the distance from the anal verge cannot affect the survival of LARC patients with radiotherapy (34). Hence, the lack of data regarding the distance between the tumor and the anus may hardly affect the scientific conclusions of this research. In addition, some recent studies indicated that chemotherapy added to radiotherapy in patients with stage II/III RC was able to reduce the risk of local recurrence but had no effect on survival (23, 24). This study failed to use local recurrence as an endpoint to discuss the decision-making of radiotherapy for rectal cancer due to the SEER database does not provide information about local recurrence. At last, detailed information regarding radiotherapy for included patients was not recorded in the SEER database. Radiotherapy for rectal cancer usually includes long-term (46–50 Gy in 23–25 fractions) and short-term (5 Gy × 5) radiotherapy. However, there is no significant survival difference between rectal cancer patients receiving long-term and short-term radiotherapy (20). This study only focused on the survival effect of radiotherapy on stage II/III RC, which to a certain extent can ignore the limitation about lacking detailed radiotherapeutic information of the SEER database. Overall, this study stated a potential correlation between radiotherapy and the age of stage II/III RC patients. However, the findings of this study still need to be further confirmed by a prospective cohort of stage II/III RC patients due to the natural limitation of the SEER database.

## Conclusion

This study indicated the inconsistent survival effect of radiotherapy on stage II/III rectal cancer patients in different age groups. Hence, we formulated a novel flow chart of radiotherapy decision-making based on age in stage II/III RC patients (**Figure 8**). Furthermore, clinicians should pay more attention to the application of radiotherapy in elderly stage II/III RC patients.

## Data Availability Statement

Publicly available datasets were analyzed in this study. This data can be found here: These data were derived from the Surveillance, Epidemiology and End Results (SEER) database (https://seer.cancer.gov/) and identified using the SEER*Stat software (Version 8.3.5) (https://seer.cancer.gov/seerstat/).

## Author Contributions

FT, HP and YL conceived and designed the study. YZ, YL and WL wrote the article. LZ downloaded and screened the data from SEER database. All authors participated in analyzing the data. All authors contributed to the article andapproved the submitted version.

## Funding

This study was supported by the following projects:i,the Natural Scientific Foundation of China (No.81702956); ii, the Natural Science Foundation of Hunan Province (No.2020JJ4903 and 2020JJ5920); iii, the Construction of Innovative Ability of National Clinical Research Center for Geriatric Disorders (No. 2019SK2335); iv, the Strategy-Oriented Special Project of Central South University of China (No. ZLXD2017003); v, the Colorectal Cancer Medical Seed Research Fund  of Beijing Bethune Public Welfare Foundation Named “Effect and mechanism of YAP1 on EGFR resistance in K-ras wild-type metastatic colorectal cancer”.

## Conflict of Interest

The authors declare that the research was conducted in the absence of any commercial or financial relationships that could be construed as a potential conflict of interest.

## Publisher’s Note

All claims expressed in this article are solely those of the authors and do not necessarily represent those of their affiliated organizations, or those of the publisher, the editors and the reviewers. Any product that may be evaluated in this article, or claim that may be made by its manufacturer, is not guaranteed or endorsed by the publisher.
