# Supplementary material for: The Survival Effect of Radiotherapy on Stage II/III Rectal Cancer in Different Age Groups: Formulating Radiotherapy Decision-Making Based on Age
Source: Front Oncol. 2021 Jul 28;11:695640. doi: 10.3389/fonc.2021.695640 (PMC8356670; doi:10.3389/fonc.2021.695640)
Supplement: Supplementary file 1 [file Image_1.pdf]

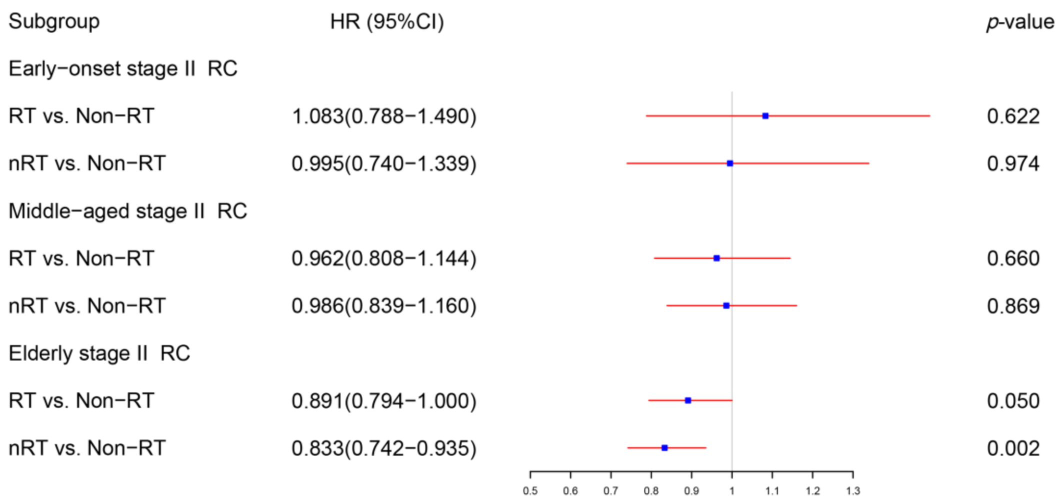

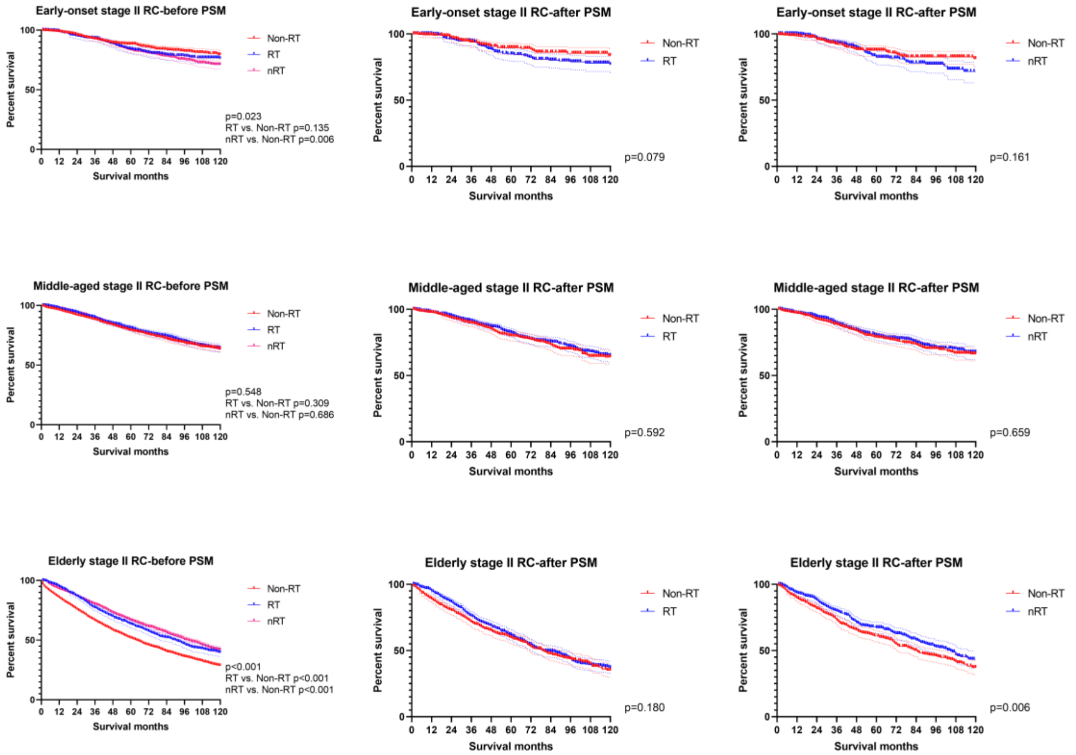

We took out the Stage II RC patients separately for analysis and got similar results comparing to the total stage II/III RC patients.

Subgroup	HR (95%CI)	*p*-value
Elderly stage II/III RC with RNE ≥12		
RT vs. Non−RT	0.974(0.897−1.058)	0.535
nRT vs. Non−RT	0.906(0.832−0.987)	0.023

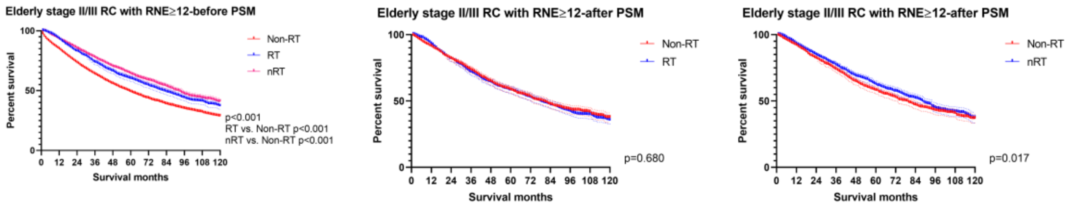

These elderly stage II/III RC patients with RNE≥12 can get survival benefit from nRT but not RT.

Subgroup	HR (95%CI)	*p*-value
Early−onset stage II/III RC with RNE< 12		
RT vs. Non−RT	1.169(0.881−1.552)	0.279
nRT vs. Non−RT	0.926(0.718−1.194)	0.553
Middle−aged stage II/III RC with RNE< 12		
RT vs. Non−RT	1.021(0.874−1.193)	0.794
nRT vs. Non−RT	0.935(0.814−1.075)	0.347

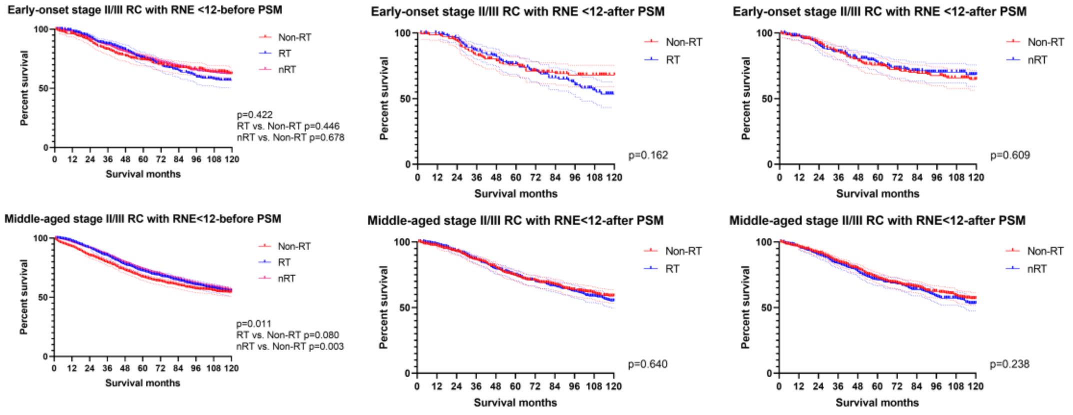

Early-onset and middle-aged stage II/III RC patients with RNE < 12 cannot obtain survival benefit from radiotherapy, including nRT and postoperative RT.
