# Supplementary material for: The Survival Effect of Radiotherapy on Stage II/III Rectal Cancer in Different Age Groups: Formulating Radiotherapy Decision-Making Based on Age
Source: Front Oncol. 2021 Jul 28;11:695640. doi: 10.3389/fonc.2021.695640 (PMC8356670; doi:10.3389/fonc.2021.695640)
Supplement: Supplementary file 2 [file Table_1.docx]

Table S1 Multivariable Cox regression model in early-onset LARC

| Characteristics | Total early-onset LARC | | | | Early-onset LARC without chemotherapy | | | |
| --- | --- | --- | --- | --- | --- | --- | --- | --- |
|  | HR | 95% CI lower | 95% CI upper | *p*-value | HR | 95% CI lower | 95% CI upper | *p*-value |
| **Radiotherapy** |  |  |  | **0.015** |  |  |  | **0.394** |
| **Non-RT** |  | **reference** |  |  |  | **reference** |  |  |
| **RT** | **1.171** | **1.017** | **1.349** | **0.028** | **1.021** | **0.669** | **1.557** | **0.925** |
| **nRT** | **0.991** | **0.870** | **1.128** | **0.887** | **0.682** | **0.391** | **1.189** | **0.177** |
| Gender |  |  |  | 0.005 |  |  |  | 0.145 |
| Female |  | reference |  |  |  | reference |  |  |
| Male | 1.145 | 1.042 | 1.259 | 0.005 | 1.215 | 0.935 | 1.579 | 0.145 |
| Marital status |  |  |  | <0.001 |  |  |  | <0.001 |
| Married |  | reference |  |  |  | reference |  |  |
| Unmarried/NOS | 1.450 | 1.321 | 1.591 | <0.001 | 1.597 | 1.229 | 2.075 | <0.001 |
| Race |  |  |  | <0.001 |  |  |  | 0.371 |
| White |  | reference |  |  |  | reference |  |  |
| Non-white | 1.264 | 1.136 | 1.406 | <0.001 | 1.142 | 0.854 | 1.526 | 0.371 |
| Pathologic grade |  |  |  | <0.001 |  |  |  | 0.012 |
| Grade I/II |  | reference |  |  |  | reference |  |  |
| Grade III/IV | 1.662 | 1.495 | 1.848 | <0.001 | 1.498 | 1.094 | 2.052 | 0.012 |
| Histologic type |  |  |  | <0.001 |  |  |  | 0.221 |
| Adenocarcinomas |  | reference |  |  |  | reference |  |  |
| MCC/SRCC | 1.552 | 1.343 | 1.794 | <0.001 | 1.364 | .830 | 2.244 | 0.221 |
| T staging |  |  |  | <0.001 |  |  |  | <0.001 |
| T1-2 |  | reference |  |  |  | reference |  |  |
| T3-4 | 1.971 | 1.649 | 2.356 | <0.001 | 2.408 | 1.472 | 3.941 | <0.001 |
| N staging |  |  |  | <0.001 |  |  |  | <0.001 |
| N0 |  | reference |  |  |  | reference |  |  |
| N+ | 1.913 | 1.712 | 2.137 | <0.001 | 3.194 | 2.407 | 4.239 | <0.001 |
| Chemotherapy |  |  |  | 0.001 |  |  |  |  |
| No |  | reference |  |  |  | NA |  |  |
| Yes | 0.764 | 0.652 | 0.896 | 0.001 |  |  |  |  |
| RNE |  |  |  | <0.001 |  |  |  | <0.001 |
| <12 |  | reference |  |  |  | reference |  |  |
| ≥12 | 0.705 | 0.637 | 0.779 | <0.001 | 0.678 | 0.504 | 0.911 | 0.010 |
| NOS | 1.483 | 1.008 | 2.181 | 0.046 | 2.738 | 1.150 | 6.518 | 0.023 |
| CEA |  |  |  | <0.001 |  |  |  | 0.038 |
| Negative |  | reference |  |  |  | reference |  |  |
| Positive | 1.478 | 1.313 | 1.662 | <0.001 | 1.520 | 1.072 | 2.157 | 0.019 |
| NOS | 1.271 | 1.136 | 1.421 | <0.001 | 1.068 | 0.784 | 1.453 | 0.678 |
| Tumor size (cm) |  |  |  | 0.008 |  |  |  | 0.207 |
| ≤5cm |  | reference |  |  |  | reference |  |  |
| > 5cm | 1.091 | 0.981 | 1.213 | 0.110 | 1.076 | 0.814 | 1.423 | 0.607 |
| NOS | 1.230 | 1.073 | 1.410 | 0.003 | 1.540 | 0.954 | 2.486 | 0.077 |
| MCC: mucinous cell carcinoma; SRCC: signet ring cell carcinoma; RNE: Regional nodes examined; nRT: Neoradiotherapy; RT: Radiotherapy (not neoadjuvant); NOS: Not otherwise specified. | | | | | | | | |

Table S2 Characteristics of early-onset LARC patients before and after PSM

| Total early-onset LARC (RT vs. Non-RT) | | | | | | | | | | |
| --- | --- | --- | --- | --- | --- | --- | --- | --- | --- | --- |
| Characteristics | Before PSM | | | | | After PSM | | | | |
|  | Non-RT (n=2444) | | RT (n=1626) | | *p*-value | Non-RT (n=1113) | | RT (n=1113) | | *p*-value |
|  | N | % | N | % |  | N | % | N | % |  |
| Gender |  |  |  |  | 0.001 |  |  |  |  | 0.832 |
| Female | 1180 | 48.3% | 696 | 42.8% |  | 514 | 46.2% | 519 | 46.6% |  |
| Male | 1264 | 51.7% | 930 | 57.2% |  | 599 | 53.8% | 594 | 53.4% |  |
| Marital status |  |  |  |  | 0.462 |  |  |  |  | 0.894 |
| Married | 1525 | 62.4% | 996 | 61.3% |  | 717 | 64.4% | 714 | 64.2% |  |
| Unmarried/NOS | 919 | 37.6% | 630 | 38.7% |  | 396 | 35.6% | 399 | 35.8% |  |
| Race |  |  |  |  | 0.878 |  |  |  |  | 0.956 |
| White | 1916 | 78.4% | 1278 | 78.6% |  | 915 | 82.2% | 914 | 82.1% |  |
| Non-white | 528 | 21.6% | 348 | 21.4% |  | 198 | 17.8% | 199 | 17.9% |  |
| Pathologic grade |  |  |  |  | 0.093 |  |  |  |  | 0.812 |
| Grade I/II | 2037 | 83.3% | 1322 | 81.3% |  | 945 | 84.9% | 949 | 85.3% |  |
| Grade III/IV | 407 | 16.7% | 304 | 18.7% |  | 168 | 15.1% | 164 | 14.7% |  |
| Histologic type |  |  |  |  | 0.090 |  |  |  |  | 0.900 |
| Adenocarcinomas | 2302 | 94.2% | 1510 | 92.9% |  | 1081 | 97.1% | 1080 | 97.0% |  |
| MCC/SRCC | 142 | 5.8% | 116 | 7.1% |  | 32 | 2.9% | 33 | 3.0% |  |
| T staging |  |  |  |  | 0.230 |  |  |  |  | 0.808 |
| T1-2 | 360 | 14.7% | 262 | 16.1% |  | 159 | 14.3% | 155 | 13.9% |  |
| T3-4 | 2084 | 85.3% | 1364 | 83.9% |  | 954 | 85.7% | 958 | 86.1% |  |
| N staging |  |  |  |  | <0.001 |  |  |  |  | 1.000 |
| N0 | 882 | 36.1% | 492 | 30.3% |  | 309 | 27.8% | 309 | 27.8% |  |
| N+ | 1562 | 63.9% | 1134 | 69.7% |  | 804 | 72.2% | 804 | 72.2% |  |
| Chemotherapy |  |  |  |  | <0.001 |  |  |  |  | 1.000 |
| No | 928 | 38.0% | 83 | 5.1% |  | 73 | 6.6% | 73 | 6.6% |  |
| Yes | 1516 | 62.0% | 1543 | 94.9% |  | 1040 | 93.4% | 1040 | 93.4% |  |
| RNE |  |  |  |  | <0.001 |  |  |  |  | 0.849 |
| <12 | 363 | 14.9% | 332 | 20.4% |  | 140 | 12.6% | 143 | 12.8% |  |
| ≥12 | 2068 | 84.6% | 1287 | 79.2% |  | 973 | 87.4% | 970 | 87.2% |  |
| NOS | 13 | 0.5% | 7 | 0.4% |  | 0 | 0.0% | 0 | 0.0% |  |
| CEA |  |  |  |  | 0.399 |  |  |  |  | 0.981 |
| Negative | 970 | 39.7% | 642 | 39.5% |  | 470 | 42.2% | 466 | 41.9% |  |
| Positive | 521 | 21.3% | 392 | 24.1% |  | 237 | 21.3% | 246 | 22.1% |  |
| NOS | 953 | 39.0% | 592 | 36.4% |  | 406 | 36.5% | 401 | 36.0% |  |
| Tumor size (cm) |  |  |  |  | 0.025 |  |  |  |  | 1.000 |
| ≤5cm | 1460 | 59.7% | 941 | 57.9% |  | 691 | 62.1% | 691 | 62.1% |  |
| > 5cm | 859 | 35.1% | 561 | 34.5% |  | 381 | 34.2% | 381 | 34.2% |  |
| NOS | 125 | 5.1% | 124 | 7.6% |  | 41 | 3.7% | 41 | 3.7% |  |
| Total early-onset LARC (nRT vs. Non-RT) | | | | | | | | | | |
| Characteristics | Before PSM | | | | | After PSM | | | | |
|  | Non-RT (n=2444) | | nRT (n=4400) | | *p*-value | Non-RT (n=1399) | | nRT (n=1399) | | *p*-value |
|  | N | % | N | % |  | N | % | N | % |  |
| Gender |  |  |  |  | <0.001 |  |  |  |  | 0.762 |
| Female | 1180 | 48.3% | 1809 | 41.1% |  | 674 | 48.2% | 666 | 47.6% |  |
| Male | 1264 | 51.7% | 2591 | 58.9% |  | 725 | 51.8% | 733 | 52.4% |  |
| Marital status |  |  |  |  | 0.091 |  |  |  |  | 0.872 |
| Married | 1525 | 62.4% | 2654 | 60.3% |  | 938 | 67.0% | 942 | 67.3% |  |
| Unmarried/NOS | 919 | 37.6% | 1746 | 39.7% |  | 461 | 33.0% | 457 | 32.7% |  |
| Race |  |  |  |  | 0.588 |  |  |  |  | 0.961 |
| White | 1916 | 78.4% | 3474 | 79.0% |  | 1138 | 81.3% | 1139 | 81.4% |  |
| Non-white | 528 | 21.6% | 926 | 21.0% |  | 261 | 18.7% | 260 | 18.6% |  |
| Pathologic grade |  |  |  |  | 0.627 |  |  |  |  | 0.389 |
| Grade I/II | 2037 | 83.3% | 3647 | 82.9% |  | 1174 | 83.9% | 1157 | 82.7% |  |
| Grade III/IV | 407 | 16.7% | 753 | 17.1% |  | 225 | 16.1% | 242 | 17.3% |  |
| Histologic type |  |  |  |  | <0.001 |  |  |  |  | 1.000 |
| Adenocarcinomas | 2302 | 94.2% | 4027 | 91.5% |  | 1331 | 95.1% | 1331 | 95.1% |  |
| MCC/SRCC | 142 | 5.8% | 373 | 8.5% |  | 68 | 4.9% | 68 | 4.9% |  |
| T staging |  |  |  |  | <0.001 |  |  |  |  | 0.864 |
| T1-2 | 360 | 14.7% | 363 | 8.3% |  | 173 | 12.4% | 176 | 12.6% |  |
| T3-4 | 2084 | 85.3% | 4037 | 91.8% |  | 1226 | 87.6% | 1223 | 87.4% |  |
| N staging |  |  |  |  | 0.004 |  |  |  |  | 0.861 |
| N0 | 882 | 36.1% | 1438 | 32.7% |  | 349 | 24.9% | 345 | 24.7% |  |
| N+ | 1562 | 63.9% | 2962 | 67.3% |  | 1050 | 75.1% | 1054 | 75.3% |  |
| Chemotherapy |  |  |  |  | <0.001 |  |  |  |  | 1.000 |
| No | 928 | 38.0% | 61 | 1.4% |  | 50 | 3.6% | 50 | 3.6% |  |
| Yes | 1516 | 62.0% | 4339 | 98.6% |  | 1349 | 96.4% | 1349 | 96.4% |  |
| RNE |  |  |  |  | <0.001 |  |  |  |  | 0.957 |
| <12 | 363 | 14.9% | 1412 | 32.1% |  | 205 | 14.7% | 204 | 14.6% |  |
| ≥12 | 2068 | 84.6% | 2939 | 66.8% |  | 1193 | 85.3% | 1194 | 85.3% |  |
| NOS | 13 | 0.5% | 49 | 1.1% |  | 1 | 0.1% | 1 | 0.1% |  |
| CEA |  |  |  |  | <0.001 |  |  |  |  | 0.914 |
| Negative | 970 | 39.7% | 1777 | 40.4% |  | 598 | 42.7% | 587 | 42.0% |  |
| Positive | 521 | 21.3% | 1292 | 29.4% |  | 316 | 22.6% | 333 | 23.8% |  |
| NOS | 953 | 39.0% | 1331 | 30.3% |  | 485 | 34.7% | 479 | 34.2% |  |
| Tumor size (cm) |  |  |  |  | <0.001 |  |  |  |  | 0.705 |
| ≤5cm | 1460 | 59.7% | 2457 | 55.8% |  | 872 | 62.3% | 882 | 63.0% |  |
| > 5cm | 859 | 35.1% | 1142 | 26.0% |  | 458 | 32.7% | 426 | 30.5% |  |
| NOS | 125 | 5.1% | 801 | 18.2% |  | 69 | 4.9% | 91 | 6.5% |  |
| Early-onset LARC without chemotherapy (RT vs. Non-RT) | | | | | | | | | | |
| Characteristics | Before PSM | | | | | After PSM | | | | |
|  | Non-RT (n=928) | | RT (n=83) | | *p*-value | Non-RT (n=69) | | RT (n=69) | | *p*-value |
|  | N | % | N | % |  | N | % | N | % |  |
| Gender |  |  |  |  | 0.831 |  |  |  |  | 0.865 |
| Female | 425 | 45.8% | 37 | 44.6% |  | 31 | 44.9% | 30 | 43.5% |  |
| Male | 503 | 54.2% | 46 | 55.4% |  | 38 | 55.1% | 39 | 56.5% |  |
| Marital status |  |  |  |  | 0.464 |  |  |  |  | 0.861 |
| Married | 543 | 58.5% | 52 | 62.7% |  | 44 | 63.8% | 43 | 62.3% |  |
| Unmarried/NOS | 385 | 41.5% | 31 | 37.3% |  | 25 | 36.2% | 26 | 37.7% |  |
| Race |  |  |  |  | 0.587 |  |  |  |  | 1.000 |
| White | 702 | 75.6% | 65 | 78.3% |  | 56 | 81.2% | 56 | 81.2% |  |
| Non-white | 226 | 24.4% | 18 | 21.7% |  | 13 | 18.8% | 13 | 18.8% |  |
| Pathologic grade |  |  |  |  | 0.413 |  |  |  |  | 1.000 |
| Grade I/II | 785 | 84.6% | 73 | 88.0% |  | 63 | 91.3% | 63 | 91.3% |  |
| Grade III/IV | 143 | 15.4% | 10 | 12.0% |  | 6 | 8.7% | 6 | 8.7% |  |
| Histologic type |  |  |  |  | 0.542 |  |  |  |  | 0.157 |
| Adenocarcinomas | 876 | 94.4% | 77 | 92.8% |  | 67 | 97.1% | 69 | 100.0% |  |
| MCC/SRCC | 52 | 5.6% | 6 | 7.2% |  | 2 | 2.9% | 0 | 0.0% |  |
| T staging |  |  |  |  | 0.288 |  |  |  |  | 0.733 |
| T1-2 | 102 | 11.0% | 6 | 7.2% |  | 5 | 7.2% | 4 | 5.8% |  |
| T3-4 | 826 | 89.0% | 77 | 92.8% |  | 64 | 92.8% | 65 | 94.2% |  |
| N staging |  |  |  |  | 0.360 |  |  |  |  | 0.866 |
| N0 | 518 | 55.8% | 42 | 50.6% |  | 38 | 55.1% | 37 | 53.6% |  |
| N+ | 410 | 44.2% | 41 | 49.4% |  | 31 | 44.9% | 32 | 46.4% |  |
| RNE |  |  |  |  | 0.080 |  |  |  |  | 1.000 |
| <12 | 148 | 15.9% | 19 | 22.9% |  | 15 | 21.7% | 15 | 21.7% |  |
| ≥12 | 773 | 83.3% | 64 | 77.1% |  | 54 | 78.3% | 54 | 78.3% |  |
| NOS | 7 | 0.8% | 0 | 0.0% |  | 0 | 0.0% | 0 | 0.0% |  |
| CEA |  |  |  |  | 0.058 |  |  |  |  | 0.921 |
| Negative | 328 | 35.3% | 21 | 25.3% |  | 18 | 26.1% | 17 | 24.6% |  |
| Positive | 193 | 20.8% | 18 | 21.7% |  | 12 | 17.4% | 15 | 21.7% |  |
| NOS | 407 | 43.9% | 44 | 53.0% |  | 39 | 56.5% | 37 | 53.6% |  |
| Tumor size (cm) |  |  |  |  | 0.997 |  |  |  |  | 0.864 |
| ≤5cm | 527 | 56.8% | 45 | 54.2% |  | 41 | 59.4% | 40 | 58.0% |  |
| > 5cm | 355 | 38.3% | 36 | 43.4% |  | 28 | 40.6% | 29 | 42.0% |  |
| NOS | 46 | 5.0% | 2 | 2.4% |  | 0 | 0.0% | 0 | 0.0% |  |
| Early-onset LARC without chemotherapy (nRT vs. Non-RT) | | | | | | | | | | |
| Characteristics | Before PSM | | | | | After PSM | | | | |
|  | Non-RT (n=928) | | nRT (n=61) | | *p*-value | Non-RT (n=48) | | nRT (n=48) | | *p*-value |
|  | N | % | N | % |  | N | % | N | % |  |
| Gender |  |  |  |  | 0.630 |  |  |  |  | 0.839 |
| Female | 425 | 45.8% | 26 | 42.6% |  | 21 | 43.8% | 20 | 41.7% |  |
| Male | 503 | 54.2% | 35 | 57.4% |  | 27 | 56.3% | 28 | 58.3% |  |
| Marital status |  |  |  |  | 0.562 |  |  |  |  | 0.834 |
| Married | 543 | 58.5% | 38 | 62.3% |  | 31 | 64.6% | 30 | 62.5% |  |
| Unmarried/NOS | 385 | 41.5% | 23 | 37.7% |  | 17 | 35.4% | 18 | 37.5% |  |
| Race |  |  |  |  | 0.158 |  |  |  |  | 0.768 |
| White | 702 | 75.6% | 51 | 83.6% |  | 42 | 87.5% | 41 | 85.4% |  |
| Non-white | 226 | 24.4% | 10 | 16.4% |  | 6 | 12.5% | 7 | 14.6% |  |
| Pathologic grade |  |  |  |  | 0.119 |  |  |  |  | 0.320 |
| Grade I/II | 785 | 84.6% | 47 | 77.0% |  | 40 | 83.3% | 36 | 75.0% |  |
| Grade III/IV | 143 | 15.4% | 14 | 23.0% |  | 8 | 16.7% | 12 | 25.0% |  |
| Histologic type |  |  |  |  | 0.173 |  |  |  |  | 0.698 |
| Adenocarcinomas | 876 | 94.4% | 55 | 90.2% |  | 45 | 93.8% | 44 | 91.7% |  |
| MCC/SRCC | 52 | 5.6% | 6 | 9.8% |  | 3 | 6.3% | 4 | 8.3% |  |
| T staging |  |  |  |  | 0.780 |  |  |  |  | 1.000 |
| T1-2 | 102 | 11.0% | 6 | 9.8% |  | 4 | 8.3% | 4 | 8.3% |  |
| T3-4 | 826 | 89.0% | 55 | 90.2% |  | 44 | 91.7% | 44 | 91.7% |  |
| N staging |  |  |  |  | 0.012 |  |  |  |  | 0.839 |
| N0 | 518 | 55.8% | 24 | 39.3% |  | 21 | 43.8% | 20 | 41.7% |  |
| N+ | 410 | 44.2% | 37 | 60.7% |  | 27 | 56.3% | 28 | 58.3% |  |
| RNE |  |  |  |  | <0.001 |  |  |  |  | 1.000 |
| <12 | 148 | 15.9% | 25 | 41.0% |  | 14 | 29.2% | 14 | 29.2% |  |
| ≥12 | 773 | 83.3% | 36 | 59.0% |  | 34 | 70.8% | 34 | 70.8% |  |
| NOS | 7 | 0.8% | 0 | 0.0% |  | 0 | 0.0% | 0 | 0.0% |  |
| CEA |  |  |  |  | 0.978 |  |  |  |  | 0.495 |
| Negative | 328 | 35.3% | 22 | 36.1% |  | 19 | 39.6% | 16 | 33.3% |  |
| Positive | 193 | 20.8% | 12 | 19.7% |  | 10 | 20.8% | 10 | 20.8% |  |
| NOS | 407 | 43.9% | 27 | 44.3% |  | 19 | 39.6% | 22 | 45.8% |  |
| Tumor size (cm) |  |  |  |  | 0.458 |  |  |  |  | 0.370 |
| ≤5cm | 527 | 56.8% | 39 | 63.9% |  | 28 | 58.3% | 34 | 70.8% |  |
| > 5cm | 355 | 38.3% | 11 | 18.0% |  | 15 | 31.3% | 9 | 18.8% |  |
| NOS | 46 | 5.0% | 11 | 18.0% |  | 5 | 10.4% | 5 | 10.4% |  |
| MCC: mucinous cell carcinoma; SRCC: signet ring cell carcinoma; RNE: Regional nodes examined; nRT: Neoradiotherapy; RT: Radiotherapy (not neoadjuvant); NOS: Not otherwise specified. | | | | | | | | | | |

Table S3 Multivariable Cox regression model in middle-aged LARC

| Characteristics | Total middle-aged LARC | | | | Middle-aged LARC without chemotherapy | | | |
| --- | --- | --- | --- | --- | --- | --- | --- | --- |
|  | HR | 95% CI lower | 95% CI upper | *p*-value | HR | 95% CI lower | 95% CI upper | *p*-value |
| **Radiotherapy** |  |  |  | **0.800** |  |  |  | **0.072** |
| **Non-RT** |  | **reference** |  |  |  | **reference** |  |  |
| **RT** | **1.010** | **0.926** | **1.102** | **0.821** | **0.892** | **0.707** | **1.125** | **0.333** |
| **nRT** | **0.985** | **0.908** | **1.068** | **0.709** | **0.704** | **0.512** | **0.969** | **0.031** |
| Gender |  |  |  | <0.001 |  |  |  | 0.001 |
| Female |  | reference |  |  |  | reference |  |  |
| Male | 1.164 | 1.096 | 1.236 | <0.001 | 1.234 | 1.084 | 1.405 | 0.001 |
| Marital status |  |  |  | <0.001 |  |  |  | <0.001 |
| Married |  | reference |  |  |  | reference |  |  |
| Unmarried/NOS | 1.356 | 1.279 | 1.437 | <0.001 | 1.492 | 1.317 | 1.689 | <0.001 |
| Race |  |  |  | 0.059 |  |  |  | 0.940 |
| White |  | reference |  |  |  | reference |  |  |
| Non-white | 1.070 | 0.998 | 1.147 | 0.059 | 0.994 | 0.858 | 1.152 | 0.940 |
| Pathologic grade |  |  |  | <0.001 |  |  |  | <0.001 |
| Grade I/II |  | reference |  |  |  | reference |  |  |
| Grade III/IV | 1.471 | 1.371 | 1.579 | <0.001 | 1.427 | 1.216 | 1.673 | <0.001 |
| Histologic type |  |  |  | <0.001 |  |  |  | 0.004 |
| Adenocarcinomas |  | reference |  |  |  | reference |  |  |
| MCC/SRCC | 1.453 | 1.322 | 1.597 | <0.001 | 1.379 | 1.105 | 1.721 | 0.004 |
| T staging |  |  |  | <0.001 |  |  |  | <0.001 |
| T1-2 |  | reference |  |  |  | reference |  |  |
| T3-4 | 1.929 | 1.725 | 2.157 | <0.001 | 2.208 | 1.718 | 2.838 | <0.001 |
| N staging |  |  |  | <0.001 |  |  |  | <0.001 |
| N0 |  | reference |  |  |  | reference |  |  |
| N+ | 1.646 | 1.545 | 1.754 | <0.001 | 2.188 | 1.921 | 2.494 | <0.001 |
| Chemotherapy |  |  |  | <0.001 |  |  |  |  |
| No |  | reference |  |  |  | NA |  |  |
| Yes | 0.741 | 0.679 | 0.809 | <0.001 |  |  |  |  |
| RNE |  |  |  | <0.001 |  |  |  | <0.001 |
| <12 |  | reference |  |  |  | reference |  |  |
| ≥12 | 0.763 | 0.718 | 0.811 | <0.001 | 0.609 | 0.532 | 0.698 | <0.001 |
| NOS | 1.361 | 1.030 | 1.799 | 0.030 | 1.191 | 0.612 | 2.315 | 0.607 |
| CEA |  |  |  | <0.001 |  |  |  | <0.001 |
| Negative |  | reference |  |  |  | reference |  |  |
| Positive | 1.623 | 1.509 | 1.746 | <0.001 | 1.694 | 1.434 | 2.001 | <0.001 |
| NOS | 1.288 | 1.200 | 1.382 | <0.001 | 1.163 | 1.001 | 1.352 | 0.049 |
| Tumor size (cm) |  |  |  | <0.001 |  |  |  | 0.002 |
| ≤5cm |  | reference |  |  |  | reference |  |  |
| > 5cm | 1.194 | 1.119 | 1.275 | <0.001 | 1.244 | 1.087 | 1.423 | 0.002 |
| NOS | 1.008 | 0.919 | 1.106 | 0.864 | 0.863 | 0.646 | 1.154 | 0.321 |
| MCC: mucinous cell carcinoma; SRCC: signet ring cell carcinoma; RNE: Regional nodes examined; nRT: Neoradiotherapy; RT: Radiotherapy (not neoadjuvant); NOS: Not otherwise specified. | | | | | | | | |

Table S4 Characteristics of middle-aged LARC patients before and after PSM

| Total middle-aged LARC (RT vs. Non-RT) | | | | | | | | | | |
| --- | --- | --- | --- | --- | --- | --- | --- | --- | --- | --- |
| Characteristics | Before PSM | | | | | After PSM | | | | |
|  | Non-RT (n=5875) | | RT (n=3510) | | *p*-value | Non-RT (n=2517) | | RT (n=2517) | | *p*-value |
|  | N | % | N | % |  | N | % | N | % |  |
| Gender |  |  |  |  | 0.020 |  |  |  |  | 0.954 |
| Female | 2416 | 41.1% | 1358 | 38.7% |  | 990 | 39.3% | 988 | 39.3% |  |
| Male | 3459 | 58.9% | 2152 | 61.3% |  | 1527 | 60.7% | 1529 | 60.7% |  |
| Marital status |  |  |  |  | 0.002 |  |  |  |  | 0.578 |
| Married | 3522 | 59.9% | 2220 | 63.2% |  | 1591 | 63.2% | 1610 | 64.0% |  |
| Unmarried/NOS | 2353 | 40.1% | 1290 | 36.8% |  | 926 | 36.8% | 907 | 36.0% |  |
| Race |  |  |  |  | 0.001 |  |  |  |  | 0.252 |
| White | 4583 | 78.0% | 2835 | 80.8% |  | 2056 | 81.7% | 2087 | 82.9% |  |
| Non-white | 1292 | 22.0% | 675 | 19.2% |  | 461 | 18.3% | 430 | 17.1% |  |
| Pathologic grade |  |  |  |  | 0.111 |  |  |  |  | 0.781 |
| Grade I/II | 4925 | 83.8% | 2898 | 82.6% |  | 2147 | 85.3% | 2140 | 85.0% |  |
| Grade III/IV | 950 | 16.2% | 612 | 17.4% |  | 370 | 14.7% | 377 | 15.0% |  |
| Histologic type |  |  |  |  | 0.012 |  |  |  |  | 0.725 |
| Adenocarcinomas | 5509 | 93.8% | 3244 | 92.4% |  | 2409 | 95.7% | 2414 | 95.9% |  |
| MCC/SRCC | 366 | 6.2% | 266 | 7.6% |  | 108 | 4.3% | 103 | 4.1% |  |
| T staging |  |  |  |  | <0.001 |  |  |  |  | 0.883 |
| T1-2 | 829 | 14.1% | 599 | 17.1% |  | 455 | 18.1% | 451 | 17.9% |  |
| T3-4 | 5046 | 85.9% | 2911 | 82.9% |  | 2062 | 81.9% | 2066 | 82.1% |  |
| N staging |  |  |  |  | <0.001 |  |  |  |  | 0.849 |
| N0 | 2465 | 42.0% | 1195 | 34.0% |  | 679 | 27.0% | 673 | 26.7% |  |
| N+ | 3410 | 58.0% | 2315 | 66.0% |  | 1838 | 73.0% | 1844 | 73.3% |  |
| Chemotherapy |  |  |  |  | <0.001 |  |  |  |  | 1.000 |
| No | 2977 | 50.7% | 239 | 6.8% |  | 220 | 8.7% | 220 | 8.7% |  |
| Yes | 2898 | 49.3% | 3271 | 93.2% |  | 2297 | 91.3% | 2297 | 91.3% |  |
| RNE |  |  |  |  | <0.001 |  |  |  |  | 0.780 |
| <12 | 1260 | 21.4% | 924 | 26.3% |  | 500 | 19.9% | 511 | 20.3% |  |
| ≥12 | 4586 | 78.1% | 2568 | 73.2% |  | 2015 | 80.1% | 2001 | 79.5% |  |
| NOS | 29 | 0.5% | 18 | 0.5% |  | 2 | 0.1% | 5 | 0.2% |  |
| CEA |  |  |  |  | 0.010 |  |  |  |  | 0.783 |
| Negative | 2125 | 36.2% | 1328 | 37.8% |  | 977 | 38.8% | 976 | 38.8% |  |
| Positive | 1382 | 23.5% | 876 | 25.0% |  | 611 | 24.3% | 596 | 23.7% |  |
| NOS | 2368 | 40.3% | 1306 | 37.2% |  | 929 | 36.9% | 945 | 37.5% |  |
| Tumor size (cm) |  |  |  |  | 0.017 |  |  |  |  | 0.556 |
| ≤5cm | 3728 | 63.5% | 2179 | 62.1% |  | 1632 | 64.8% | 1654 | 65.7% |  |
| > 5cm | 1847 | 31.4% | 1093 | 31.1% |  | 769 | 30.6% | 749 | 29.8% |  |
| NOS | 300 | 5.1% | 238 | 6.8% |  | 116 | 4.6% | 114 | 4.5% |  |
| Total middle-aged LARC (nRT vs. Non-RT) | | | | | | | | | | |
| Characteristics | Before PSM | | | | | After PSM | | | | |
|  | Non-RT (n=5875) | | nRT (n=8327) | | *p*-value | Non-RT (n=2697) | | nRT (n=2697) | | *p*-value |
|  | N | % | N | % |  | N | % | N | % |  |
| Gender |  |  |  |  | <0.001 |  |  |  |  | 1.000 |
| Female | 2416 | 41.1% | 2917 | 35.0% |  | 1075 | 39.9% | 1075 | 39.9% |  |
| Male | 3459 | 58.9% | 5410 | 65.0% |  | 1622 | 60.1% | 1622 | 60.1% |  |
| Marital status |  |  |  |  | 0.133 |  |  |  |  | 0.652 |
| Married | 3522 | 59.9% | 5096 | 61.2% |  | 1709 | 63.4% | 1693 | 62.8% |  |
| Unmarried/NOS | 2353 | 40.1% | 3231 | 38.8% |  | 988 | 36.6% | 1004 | 37.2% |  |
| Race |  |  |  |  | 0.002 |  |  |  |  | 0.681 |
| White | 4583 | 78.0% | 6677 | 80.2% |  | 2174 | 80.6% | 2162 | 80.2% |  |
| Non-white | 1292 | 22.0% | 1650 | 19.8% |  | 523 | 19.4% | 535 | 19.8% |  |
| Pathologic grade |  |  |  |  | <0.001 |  |  |  |  | 0.880 |
| Grade I/II | 4925 | 83.8% | 7185 | 86.3% |  | 2284 | 84.7% | 2280 | 84.5% |  |
| Grade III/IV | 950 | 16.2% | 1142 | 13.7% |  | 413 | 15.3% | 417 | 15.5% |  |
| Histologic type |  |  |  |  | 0.313 |  |  |  |  | 0.801 |
| Adenocarcinomas | 5509 | 93.8% | 7773 | 93.3% |  | 2562 | 95.0% | 2566 | 95.1% |  |
| MCC/SRCC | 366 | 6.2% | 554 | 6.7% |  | 135 | 5.0% | 131 | 4.9% |  |
| T staging |  |  |  |  | <0.001 |  |  |  |  | 0.968 |
| T1-2 | 829 | 14.1% | 598 | 7.2% |  | 354 | 13.1% | 355 | 13.2% |  |
| T3-4 | 5046 | 85.9% | 7729 | 92.8% |  | 2343 | 86.9% | 2342 | 86.8% |  |
| N staging |  |  |  |  | 0.032 |  |  |  |  | 0.900 |
| N0 | 2465 | 42.0% | 3344 | 40.2% |  | 674 | 25.0% | 670 | 24.8% |  |
| N+ | 3410 | 58.0% | 4983 | 59.8% |  | 2023 | 75.0% | 2027 | 75.2% |  |
| Chemotherapy |  |  |  |  | <0.001 |  |  |  |  | 1.000 |
| No | 2977 | 50.7% | 150 | 1.8% |  | 121 | 4.5% | 121 | 4.5% |  |
| Yes | 2898 | 49.3% | 8177 | 98.2% |  | 2576 | 95.5% | 2576 | 95.5% |  |
| RNE |  |  |  |  | <0.001 |  |  |  |  | 0.816 |
| <12 | 1260 | 21.4% | 3216 | 38.6% |  | 562 | 20.8% | 557 | 20.7% |  |
| ≥12 | 4586 | 78.1% | 5046 | 60.6% |  | 2131 | 79.0% | 2134 | 79.1% |  |
| NOS | 29 | 0.5% | 65 | 0.8% |  | 4 | 0.1% | 6 | 0.2% |  |
| CEA |  |  |  |  | <0.001 |  |  |  |  | 0.850 |
| Negative | 2125 | 36.2% | 3141 | 37.7% |  | 1052 | 39.0% | 1044 | 38.7% |  |
| Positive | 1382 | 23.5% | 2656 | 31.9% |  | 693 | 25.7% | 697 | 25.8% |  |
| NOS | 2368 | 40.3% | 2530 | 30.4% |  | 952 | 35.3% | 956 | 35.4% |  |
| Tumor size (cm) |  |  |  |  | <0.001 |  |  |  |  | 0.584 |
| ≤5cm | 3728 | 63.5% | 4703 | 56.5% |  | 1787 | 66.3% | 1777 | 65.9% |  |
| > 5cm | 1847 | 31.4% | 2073 | 24.9% |  | 759 | 28.1% | 755 | 28.0% |  |
| NOS | 300 | 5.1% | 1551 | 18.6% |  | 151 | 5.6% | 165 | 6.1% |  |
| Middle-aged LARC without chemotherapy (RT vs. Non-RT) | | | | | | | | | | |
| Characteristics | Before PSM | | | | | After PSM | | | | |
|  | Non-RT (n=2977) | | RT (n=239) | | *p*-value | Non-RT (n=217) | | RT (n=217) | | *p*-value |
|  | N | % | N | % |  | N | % | N | % |  |
| Gender |  |  |  |  | 0.469 |  |  |  |  | 0.920 |
| Female | 1192 | 40.0% | 90 | 37.7% |  | 75 | 34.6% | 76 | 35.0% |  |
| Male | 1785 | 60.0% | 149 | 62.3% |  | 142 | 65.4% | 141 | 65.0% |  |
| Marital status |  |  |  |  | 0.201 |  |  |  |  | 0.692 |
| Married | 1692 | 56.8% | 146 | 61.1% |  | 138 | 63.6% | 134 | 61.8% |  |
| Unmarried/NOS | 1285 | 43.2% | 93 | 38.9% |  | 79 | 36.4% | 83 | 38.2% |  |
| Race |  |  |  |  | 0.432 |  |  |  |  | 0.904 |
| White | 2288 | 76.9% | 189 | 79.1% |  | 174 | 80.2% | 175 | 80.6% |  |
| Non-white | 689 | 23.1% | 50 | 20.9% |  | 43 | 19.8% | 42 | 19.4% |  |
| Pathologic grade |  |  |  |  | 0.131 |  |  |  |  | 0.884 |
| Grade I/II | 2571 | 86.4% | 198 | 82.8% |  | 190 | 87.6% | 191 | 88.0% |  |
| Grade III/IV | 406 | 13.6% | 41 | 17.2% |  | 27 | 12.4% | 26 | 12.0% |  |
| Histologic type |  |  |  |  | 0.689 |  |  |  |  | 0.503 |
| Adenocarcinomas | 2796 | 93.9% | 226 | 94.6% |  | 205 | 94.5% | 208 | 95.9% |  |
| MCC/SRCC | 181 | 6.1% | 13 | 5.4% |  | 12 | 5.5% | 9 | 4.1% |  |
| T staging |  |  |  |  | 0.174 |  |  |  |  | 0.298 |
| T1-2 | 292 | 9.8% | 30 | 12.6% |  | 29 | 13.4% | 22 | 10.1% |  |
| T3-4 | 2685 | 90.2% | 209 | 87.4% |  | 188 | 86.6% | 195 | 89.9% |  |
| N staging |  |  |  |  | 0.001 |  |  |  |  | 0.848 |
| N0 | 1831 | 61.5% | 122 | 51.0% |  | 115 | 53.0% | 117 | 53.9% |  |
| N+ | 1146 | 38.5% | 117 | 49.0% |  | 102 | 47.0% | 100 | 46.1% |  |
| RNE |  |  |  |  | 0.004 |  |  |  |  | 0.832 |
| <12 | 688 | 23.1% | 75 | 31.4% |  | 61 | 28.1% | 63 | 29.0% |  |
| ≥12 | 2274 | 76.4% | 163 | 68.2% |  | 156 | 71.9% | 154 | 71.0% |  |
| NOS | 15 | 0.5% | 1 | 0.4% |  | 0 | 0.0% | 0 | 0.0% |  |
| CEA |  |  |  |  | 0.436 |  |  |  |  | 1.000 |
| Negative | 986 | 33.1% | 79 | 33.1% |  | 73 | 33.6% | 72 | 33.2% |  |
| Positive | 668 | 22.4% | 43 | 18.0% |  | 38 | 17.5% | 40 | 18.4% |  |
| NOS | 1323 | 44.4% | 117 | 49.0% |  | 106 | 48.8% | 105 | 48.4% |  |
| Tumor size (cm) |  |  |  |  | 0.790 |  |  |  |  | 0.936 |
| ≤5cm | 1799 | 60.4% | 149 | 62.3% |  | 138 | 63.6% | 137 | 63.1% |  |
| > 5cm | 1042 | 35.0% | 72 | 30.1% |  | 67 | 30.9% | 68 | 31.3% |  |
| NOS | 136 | 4.6% | 18 | 7.5% |  | 12 | 5.5% | 12 | 5.5% |  |
| Middle-aged LARC without chemotherapy (nRT vs. Non-RT) | | | | | | | | | | |
| Characteristics | Before PSM | | | | | After PSM | | | | |
|  | Non-RT (n=2977) | | nRT (n=150) | | *p*-value | Non-RT (n=118) | | nRT (n=118) | | *p*-value |
|  | N | % | N | % |  | N | % | N | % |  |
| Gender |  |  |  |  | 0.140 |  |  |  |  | 0.684 |
| Female | 1192 | 40.0% | 51 | 34.0% |  | 40 | 33.9% | 43 | 36.4% |  |
| Male | 1785 | 60.0% | 99 | 66.0% |  | 78 | 66.1% | 75 | 63.6% |  |
| Marital status |  |  |  |  | 0.398 |  |  |  |  | 0.602 |
| Married | 1692 | 56.8% | 80 | 53.3% |  | 67 | 56.8% | 63 | 53.4% |  |
| Unmarried/NOS | 1285 | 43.2% | 70 | 46.7% |  | 51 | 43.2% | 55 | 46.6% |  |
| Race |  |  |  |  | 0.119 |  |  |  |  | 0.766 |
| White | 2288 | 76.9% | 107 | 71.3% |  | 87 | 73.7% | 89 | 75.4% |  |
| Non-white | 689 | 23.1% | 43 | 28.7% |  | 31 | 26.3% | 29 | 24.6% |  |
| Pathologic grade |  |  |  |  | 0.008 |  |  |  |  | 0.735 |
| Grade I/II | 2571 | 86.4% | 118 | 78.7% |  | 98 | 83.1% | 96 | 81.4% |  |
| Grade III/IV | 406 | 13.6% | 32 | 21.3% |  | 20 | 16.9% | 22 | 18.6% |  |
| Histologic type |  |  |  |  | 0.533 |  |  |  |  | 1.000 |
| Adenocarcinomas | 2796 | 93.9% | 139 | 92.7% |  | 115 | 97.5% | 115 | 97.5% |  |
| MCC/SRCC | 181 | 6.1% | 11 | 7.3% |  | 3 | 2.5% | 3 | 2.5% |  |
| T staging |  |  |  |  | 0.123 |  |  |  |  | 0.606 |
| T1-2 | 292 | 9.8% | 9 | 6.0% |  | 9 | 7.6% | 7 | 5.9% |  |
| T3-4 | 2685 | 90.2% | 141 | 94.0% |  | 109 | 92.4% | 111 | 94.1% |  |
| N staging |  |  |  |  | 0.045 |  |  |  |  | 1.000 |
| N0 | 1831 | 61.5% | 80 | 53.3% |  | 66 | 55.9% | 66 | 55.9% |  |
| N+ | 1146 | 38.5% | 70 | 46.7% |  | 52 | 44.1% | 52 | 44.1% |  |
| RNE |  |  |  |  | <0.001 |  |  |  |  | 0.436 |
| <12 | 688 | 23.1% | 65 | 43.3% |  | 46 | 39.0% | 42 | 35.6% |  |
| ≥12 | 2274 | 76.4% | 81 | 54.0% |  | 72 | 61.0% | 74 | 62.7% |  |
| NOS | 15 | 0.5% | 4 | 2.7% |  | 0 | 0.0% | 2 | 1.7% |  |
| CEA |  |  |  |  | 0.362 |  |  |  |  | 0.823 |
| Negative | 986 | 33.1% | 52 | 34.7% |  | 42 | 35.6% | 43 | 36.4% |  |
| Positive | 668 | 22.4% | 39 | 26.0% |  | 28 | 23.7% | 29 | 24.6% |  |
| NOS | 1323 | 44.4% | 59 | 39.3% |  | 48 | 40.7% | 46 | 39.0% |  |
| Tumor size (cm) |  |  |  |  | <0.001 |  |  |  |  | 0.651 |
| ≤5cm | 1799 | 60.4% | 82 | 54.7% |  | 72 | 61.0% | 72 | 61.0% |  |
| > 5cm | 1042 | 35.0% | 30 | 20.0% |  | 33 | 28.0% | 28 | 23.7% |  |
| NOS | 136 | 4.6% | 38 | 25.3% |  | 13 | 11.0% | 18 | 15.3% |  |
| MCC: mucinous cell carcinoma; SRCC: signet ring cell carcinoma; RNE: Regional nodes examined; nRT: Neoradiotherapy; RT: Radiotherapy (not neoadjuvant); NOS: Not otherwise specified. | | | | | | | | | | |

Table S5 Multivariable Cox regression model in elderly LARC

| Characteristics | Total elderly LARC | | | | Elderly LARC without chemotherapy | | | |
| --- | --- | --- | --- | --- | --- | --- | --- | --- |
|  | HR | 95% CI lower | 95% CI upper | *p*-value | HR | 95% CI lower | 95% CI upper | *p*-value |
| **Radiotherapy** |  |  |  | **<0.001** |  |  |  | **<0.001** |
| **Non-RT** |  | **reference** |  |  |  | **reference** |  |  |
| **RT** | **0.942** | **0.882** | **1.005** | **0.070** | **0.808** | **0.715** | **0.913** | **0.001** |
| **nRT** | **0.866** | **0.812** | **0.924** | **<0.001** | **0.729** | **0.611** | **0.871** | **<0.001** |
| Gender |  |  |  | <0.001 |  |  |  | <0.001 |
| Female |  | reference |  |  |  | reference |  |  |
| Male | 1.223 | 1.173 | 1.275 | <0.001 | 1.242 | 1.173 | 1.315 | <0.001 |
| Marital status |  |  |  | <0.001 |  |  |  | <0.001 |
| Married |  | reference |  |  |  | reference |  |  |
| Unmarried/NOS | 1.337 | 1.282 | 1.394 | <0.001 | 1.336 | 1.262 | 1.415 | <0.001 |
| Race |  |  |  | 0.005 |  |  |  | 0.002 |
| White |  | reference |  |  |  | reference |  |  |
| Non-white | 0.926 | 0.878 | 0.977 | 0.005 | 0.890 | 0.827 | 0.957 | 0.002 |
| Pathologic grade |  |  |  | <0.001 |  |  |  | <0.001 |
| Grade I/II |  | reference |  |  |  | reference |  |  |
| Grade III/IV | 1.333 | 1.268 | 1.401 | <0.001 | 1.344 | 1.255 | 1.439 | <0.001 |
| Histologic type |  |  |  | <0.001 |  |  |  | 0.008 |
| Adenocarcinomas |  | reference |  |  |  | reference |  |  |
| MCC/SRCC | 1.270 | 1.186 | 1.359 | <0.001 | 1.143 | 1.036 | 1.262 | 0.008 |
| T staging |  |  |  | <0.001 |  |  |  | <0.001 |
| T1-2 |  | reference |  |  |  | reference |  |  |
| T3-4 | 1.517 | 1.408 | 1.634 | <0.001 | 1.512 | 1.363 | 1.677 | <0.001 |
| N staging |  |  |  | <0.001 |  |  |  | <0.001 |
| N0 |  | reference |  |  |  | reference |  |  |
| N+ | 1.527 | 1.463 | 1.594 | <0.001 | 1.689 | 1.595 | 1.789 | <0.001 |
| Chemotherapy |  |  |  | <0.001 |  |  |  |  |
| No |  | reference |  |  |  | NA |  |  |
| Yes | 0.573 | 0.542 | 0.606 | <0.001 |  |  |  |  |
| RNE |  |  |  | <0.001 |  |  |  | <0.001 |
| <12 |  | reference |  |  |  | reference |  |  |
| ≥12 | 0.811 | 0.778 | 0.845 | <0.001 | 0.792 | 0.749 | 0.838 | <0.001 |
| NOS | 1.273 | 1.037 | 1.563 | 0.021 | 1.133 | 0.827 | 1.552 | .437 |
| CEA |  |  |  | <0.001 |  |  |  | <0.001 |
| Negative |  | reference |  |  |  | reference |  |  |
| Positive | 1.394 | 1.323 | 1.470 | <0.001 | 1.404 | 1.304 | 1.512 | <0.001 |
| NOS | 1.246 | 1.188 | 1.307 | <0.001 | 1.225 | 1.147 | 1.308 | <0.001 |
| Tumor size (cm) |  |  |  | <0.001 |  |  |  | 0.001 |
| ≤5cm |  | reference |  |  |  | reference |  |  |
| > 5cm | 1.135 | 1.086 | 1.187 | <0.001 | 1.121 | 1.057 | 1.189 | <0.001 |
| NOS | 1.107 | 1.029 | 1.192 | 0.007 | 1.084 | 0.951 | 1.236 | 0.229 |
| MCC: mucinous cell carcinoma; SRCC: signet ring cell carcinoma; RNE: Regional nodes examined; nRT: Neoradiotherapy; RT: Radiotherapy (not neoadjuvant); NOS: Not otherwise specified. | | | | | | | | |

Table S6 Characteristics of elderly LARC patients before and after PSM

| Total elderly LARC (RT vs. Non-RT) | | | | | | | | | | |
| --- | --- | --- | --- | --- | --- | --- | --- | --- | --- | --- |
| Characteristics | Before PSM | | | | | After PSM | | | | |
|  | Non-RT (n=10495) | | RT (n=3013) | | *p*-value | Non-RT (n=2306) | | RT (n=2306) | | *p*-value |
|  | N | % | N | % |  | N | % | N | % |  |
| Gender |  |  |  |  | <0.001 |  |  |  |  | 0.953 |
| Female | 4847 | 46.2% | 1280 | 42.5% |  | 985 | 42.7% | 983 | 42.6% |  |
| Male | 5648 | 53.8% | 1733 | 57.5% |  | 1321 | 57.3% | 1323 | 57.4% |  |
| Marital status |  |  |  |  | <0.001 |  |  |  |  | 0.299 |
| Married | 5173 | 49.3% | 1760 | 58.4% |  | 1286 | 55.8% | 1321 | 57.3% |  |
| Unmarried/NOS | 5322 | 50.7% | 1253 | 41.6% |  | 1020 | 44.2% | 985 | 42.7% |  |
| Race |  |  |  |  | 0.137 |  |  |  |  | 0.206 |
| White | 8681 | 82.7% | 2457 | 81.5% |  | 1907 | 82.7% | 1939 | 84.1% |  |
| Non-white | 1814 | 17.3% | 556 | 18.5% |  | 399 | 17.3% | 367 | 15.9% |  |
| Pathologic grade |  |  |  |  | 0.007 |  |  |  |  | 0.426 |
| Grade I/II | 8675 | 82.7% | 2426 | 80.5% |  | 1869 | 81.0% | 1890 | 82.0% |  |
| Grade III/IV | 1820 | 17.3% | 587 | 19.5% |  | 437 | 19.0% | 416 | 18.0% |  |
| Histologic type |  |  |  |  | 0.832 |  |  |  |  | 0.222 |
| Adenocarcinomas | 9706 | 92.5% | 2783 | 92.4% |  | 2137 | 92.7% | 2158 | 93.6% |  |
| MCC/SRCC | 789 | 7.5% | 230 | 7.6% |  | 169 | 7.3% | 148 | 6.4% |  |
| T staging |  |  |  |  | <0.001 |  |  |  |  | 0.966 |
| T1-2 | 1067 | 10.2% | 398 | 13.2% |  | 309 | 13.4% | 308 | 13.4% |  |
| T3-4 | 9428 | 89.8% | 2615 | 86.8% |  | 1997 | 86.6% | 1998 | 86.6% |  |
| N staging |  |  |  |  | <0.001 |  |  |  |  | 0.923 |
| N0 | 5222 | 49.8% | 1131 | 37.5% |  | 698 | 30.3% | 701 | 30.4% |  |
| N+ | 5273 | 50.2% | 1882 | 62.5% |  | 1608 | 69.7% | 1605 | 69.6% |  |
| Chemotherapy |  |  |  |  | <0.001 |  |  |  |  | 1.000 |
| No | 8003 | 76.3% | 430 | 14.3% |  | 423 | 18.3% | 423 | 18.3% |  |
| Yes | 2492 | 23.7% | 2583 | 85.7% |  | 1883 | 81.7% | 1883 | 81.7% |  |
| RNE |  |  |  |  | 0.028 |  |  |  |  | 0.697 |
| <12 | 3150 | 30.0% | 980 | 32.5% |  | 636 | 27.6% | 632 | 27.4% |  |
| ≥12 | 7286 | 69.4% | 2005 | 66.5% |  | 1665 | 72.2% | 1661 | 72.0% |  |
| NOS | 59 | 0.6% | 28 | 0.9% |  | 5 | 0.2% | 13 | 0.6% |  |
| CEA |  |  |  |  | 0.001 |  |  |  |  | 0.707 |
| Negative | 3277 | 31.2% | 1043 | 34.6% |  | 823 | 35.7% | 833 | 36.1% |  |
| Positive | 2686 | 25.6% | 744 | 24.7% |  | 612 | 26.5% | 570 | 24.7% |  |
| NOS | 4532 | 43.2% | 1226 | 40.7% |  | 871 | 37.8% | 903 | 39.2% |  |
| Tumor size (cm) |  |  |  |  | 0.313 |  |  |  |  | 0.874 |
| ≤5cm | 6699 | 63.8% | 1915 | 63.6% |  | 1528 | 66.3% | 1519 | 65.9% |  |
| > 5cm | 3360 | 32.0% | 945 | 31.4% |  | 685 | 29.7% | 709 | 30.7% |  |
| NOS | 436 | 4.2% | 153 | 5.1% |  | 93 | 4.0% | 78 | 3.4% |  |
| Total elderly LARC (nRT vs. Non-RT) | | | | | | | | | | |
| Characteristics | Before PSM | | | | | After PSM | | | | |
|  | Non-RT (n=10495) | | nRT (n=6154) | | *p*-value | Non-RT (n=2360) | | nRT (n=2360) | | *p*-value |
|  | N | % | N | % |  | N | % | N | % |  |
| Gender |  |  |  |  | <0.001 |  |  |  |  | 0.790 |
| Female | 4847 | 46.2% | 2402 | 39.0% |  | 972 | 41.2% | 963 | 40.8% |  |
| Male | 5648 | 53.8% | 3752 | 61.0% |  | 1388 | 58.8% | 1397 | 59.2% |  |
| Marital status |  |  |  |  | <0.001 |  |  |  |  | 0.906 |
| Married | 5173 | 49.3% | 3595 | 58.4% |  | 1365 | 57.8% | 1369 | 58.0% |  |
| Unmarried/NOS | 5322 | 50.7% | 2559 | 41.6% |  | 995 | 42.2% | 991 | 42.0% |  |
| Race |  |  |  |  | 0.101 |  |  |  |  | 0.683 |
| White | 8681 | 82.7% | 5151 | 83.7% |  | 2003 | 84.9% | 2013 | 85.3% |  |
| Non-white | 1814 | 17.3% | 1003 | 16.3% |  | 357 | 15.1% | 347 | 14.7% |  |
| Pathologic grade |  |  |  |  | <0.001 |  |  |  |  | 0.969 |
| Grade I/II | 8675 | 82.7% | 5296 | 86.1% |  | 1954 | 82.8% | 1955 | 82.8% |  |
| Grade III/IV | 1820 | 17.3% | 858 | 13.9% |  | 406 | 17.2% | 405 | 17.2% |  |
| Histologic type |  |  |  |  | 0.299 |  |  |  |  | 0.653 |
| Adenocarcinomas | 9706 | 92.5% | 5664 | 92.0% |  | 2194 | 93.0% | 2186 | 92.6% |  |
| MCC/SRCC | 789 | 7.5% | 490 | 8.0% |  | 166 | 7.0% | 174 | 7.4% |  |
| T staging |  |  |  |  | <0.001 |  |  |  |  | 0.887 |
| T1-2 | 1067 | 10.2% | 398 | 6.5% |  | 251 | 10.6% | 248 | 10.5% |  |
| T3-4 | 9428 | 89.8% | 5756 | 93.5% |  | 2109 | 89.4% | 2112 | 89.5% |  |
| N staging |  |  |  |  | <0.001 |  |  |  |  | 0.496 |
| N0 | 5222 | 49.8% | 2874 | 46.7% |  | 672 | 28.5% | 651 | 27.6% |  |
| N+ | 5273 | 50.2% | 3280 | 53.3% |  | 1688 | 71.5% | 1709 | 72.4% |  |
| Chemotherapy |  |  |  |  | <0.001 |  |  |  |  | 1.000 |
| No | 8003 | 76.3% | 241 | 3.9% |  | 233 | 9.9% | 233 | 9.9% |  |
| Yes | 2492 | 23.7% | 5913 | 96.1% |  | 2127 | 90.1% | 2127 | 90.1% |  |
| RNE |  |  |  |  | <0.001 |  |  |  |  | 0.924 |
| <12 | 3150 | 30.0% | 2745 | 44.6% |  | 672 | 28.5% | 677 | 28.7% |  |
| ≥12 | 7286 | 69.4% | 3360 | 54.6% |  | 1682 | 71.3% | 1675 | 71.0% |  |
| NOS | 59 | 0.6% | 49 | 0.8% |  | 6 | 0.3% | 8 | 0.3% |  |
| CEA |  |  |  |  | <0.001 |  |  |  |  | 0.986 |
| Negative | 3277 | 31.2% | 2126 | 34.5% |  | 839 | 35.6% | 838 | 35.5% |  |
| Positive | 2686 | 25.6% | 1864 | 30.3% |  | 676 | 28.6% | 677 | 28.7% |  |
| NOS | 4532 | 43.2% | 2164 | 35.2% |  | 845 | 35.8% | 845 | 35.8% |  |
| Tumor size (cm) |  |  |  |  | <0.001 |  |  |  |  | 0.701 |
| ≤5cm | 6699 | 63.8% | 3574 | 58.1% |  | 1544 | 65.4% | 1556 | 65.9% |  |
| > 5cm | 3360 | 32.0% | 1407 | 22.9% |  | 682 | 28.9% | 642 | 27.2% |  |
| NOS | 436 | 4.2% | 1173 | 19.1% |  | 134 | 5.7% | 162 | 6.9% |  |
| Elderly LARC without chemotherapy (RT vs. Non-RT) | | | | | | | | | | |
| Characteristics | Before PSM | | | | | After PSM | | | | |
|  | Non-RT (n=8003) | | RT (n=430) | | *p*-value | Non-RT (n=417) | | RT (n=417) | | *p*-value |
|  | N | % | N | % |  | N | % | N | % |  |
| Gender |  |  |  |  | 0.050 |  |  |  |  | 0.624 |
| Female | 3774 | 47.2% | 182 | 42.3% |  | 172 | 41.2% | 179 | 42.9% |  |
| Male | 4229 | 52.8% | 248 | 57.7% |  | 245 | 58.8% | 238 | 57.1% |  |
| Marital status |  |  |  |  | 0.012 |  |  |  |  | 0.677 |
| Married | 3748 | 46.8% | 228 | 53.0% |  | 228 | 54.7% | 222 | 53.2% |  |
| Unmarried/NOS | 4255 | 53.2% | 202 | 47.0% |  | 189 | 45.3% | 195 | 46.8% |  |
| Race |  |  |  |  | 0.046 |  |  |  |  | 0.182 |
| White | 6627 | 82.8% | 340 | 79.1% |  | 317 | 76.0% | 333 | 79.9% |  |
| Non-white | 1376 | 17.2% | 90 | 20.9% |  | 100 | 24.0% | 84 | 20.1% |  |
| Pathologic grade |  |  |  |  | 0.012 |  |  |  |  | 0.663 |
| Grade I/II | 6699 | 83.7% | 340 | 79.1% |  | 338 | 81.1% | 333 | 79.9% |  |
| Grade III/IV | 1304 | 16.3% | 90 | 20.9% |  | 79 | 18.9% | 84 | 20.1% |  |
| Histologic type |  |  |  |  | 0.949 |  |  |  |  | 0.887 |
| Adenocarcinomas | 7414 | 92.6% | 398 | 92.6% |  | 391 | 93.8% | 390 | 93.5% |  |
| MCC/SRCC | 589 | 7.4% | 32 | 7.4% |  | 26 | 6.2% | 27 | 6.5% |  |
| T staging |  |  |  |  | 0.431 |  |  |  |  | 0.717 |
| T1-2 | 676 | 8.4% | 41 | 9.5% |  | 36 | 8.6% | 39 | 9.4% |  |
| T3-4 | 7327 | 91.6% | 389 | 90.5% |  | 381 | 91.4% | 378 | 90.6% |  |
| N staging |  |  |  |  | <0.001 |  |  |  |  | 0.782 |
| N0 | 4650 | 58.1% | 211 | 49.1% |  | 208 | 49.9% | 204 | 48.9% |  |
| N+ | 3353 | 41.9% | 219 | 50.9% |  | 209 | 50.1% | 213 | 51.1% |  |
| RNE |  |  |  |  | 0.202 |  |  |  |  | 0.393 |
| <12 | 2523 | 31.5% | 151 | 35.1% |  | 132 | 31.7% | 145 | 34.8% |  |
| ≥12 | 5434 | 67.9% | 274 | 63.7% |  | 282 | 67.6% | 268 | 64.3% |  |
| NOS | 46 | 0.6% | 5 | 1.2% |  | 3 | 0.7% | 4 | 1.0% |  |
| CEA |  |  |  |  | 0.860 |  |  |  |  | 0.873 |
| Negative | 2353 | 29.4% | 133 | 30.9% |  | 126 | 30.2% | 130 | 31.2% |  |
| Positive | 2009 | 25.1% | 98 | 22.8% |  | 96 | 23.0% | 92 | 22.1% |  |
| NOS | 3641 | 45.5% | 199 | 46.3% |  | 195 | 46.8% | 195 | 46.8% |  |
| Tumor size (cm) |  |  |  |  | 0.029 |  |  |  |  | 0.574 |
| ≤5cm | 5029 | 62.8% | 249 | 57.9% |  | 246 | 59.0% | 244 | 58.5% |  |
| > 5cm | 2662 | 33.3% | 159 | 37.0% |  | 162 | 38.8% | 157 | 37.6% |  |
| NOS | 312 | 3.9% | 22 | 5.1% |  | 9 | 2.2% | 16 | 3.8% |  |
| Elderly LARC without chemotherapy (nRT vs. Non-RT) | | | | | | | | | | |
| Characteristics | Before PSM | | | | | After PSM | | | | |
|  | Non-RT (n=8003) | | nRT (n=241) | | *p*-value | Non-RT (n=231) | | nRT (n=231) | | *p*-value |
|  | N | % | N | % |  | N | % | N | % |  |
| Gender |  |  |  |  | 0.964 |  |  |  |  | 0.926 |
| Female | 3774 | 47.2% | 114 | 47.3% |  | 112 | 48.5% | 111 | 48.1% |  |
| Male | 4229 | 52.8% | 127 | 52.7% |  | 119 | 51.5% | 120 | 51.9% |  |
| Marital status |  |  |  |  | 0.029 |  |  |  |  | 0.926 |
| Married | 3748 | 46.8% | 130 | 53.9% |  | 122 | 52.8% | 121 | 52.4% |  |
| Unmarried/NOS | 4255 | 53.2% | 111 | 46.1% |  | 109 | 47.2% | 110 | 47.6% |  |
| Race |  |  |  |  | 0.443 |  |  |  |  | 0.902 |
| White | 6627 | 82.8% | 195 | 80.9% |  | 192 | 83.1% | 191 | 82.7% |  |
| Non-white | 1376 | 17.2% | 46 | 19.1% |  | 39 | 16.9% | 40 | 17.3% |  |
| Pathologic grade |  |  |  |  | 0.766 |  |  |  |  | 1.000 |
| Grade I/II | 6699 | 83.7% | 200 | 83.0% |  | 192 | 83.1% | 192 | 83.1% |  |
| Grade III/IV | 1304 | 16.3% | 41 | 17.0% |  | 39 | 16.9% | 39 | 16.9% |  |
| Histologic type |  |  |  |  | 0.949 |  |  |  |  | 0.608 |
| Adenocarcinomas | 7414 | 92.6% | 223 | 92.5% |  | 211 | 91.3% | 214 | 92.6% |  |
| MCC/SRCC | 589 | 7.4% | 18 | 7.5% |  | 20 | 8.7% | 17 | 7.4% |  |
| T staging |  |  |  |  | 0.055 |  |  |  |  | 0.687 |
| T1-2 | 676 | 8.4% | 12 | 5.0% |  | 14 | 6.1% | 12 | 5.2% |  |
| T3-4 | 7327 | 91.6% | 229 | 95.0% |  | 217 | 93.9% | 219 | 94.8% |  |
| N staging |  |  |  |  | 0.001 |  |  |  |  | 0.516 |
| N0 | 4650 | 58.1% | 114 | 47.3% |  | 117 | 50.6% | 110 | 47.6% |  |
| N+ | 3353 | 41.9% | 127 | 52.7% |  | 114 | 49.4% | 121 | 52.4% |  |
| RNE |  |  |  |  | <0.001 |  |  |  |  | 0.852 |
| <12 | 2523 | 31.5% | 109 | 45.2% |  | 106 | 45.9% | 104 | 45.0% |  |
| ≥12 | 5434 | 67.9% | 131 | 54.4% |  | 125 | 54.1% | 127 | 55.0% |  |
| NOS | 46 | 0.6% | 1 | 0.4% |  | 0 | 0.0% | 0 | 0.0% |  |
| CEA |  |  |  |  | 0.753 |  |  |  |  | 1.000 |
| Negative | 2353 | 29.4% | 64 | 26.6% |  | 62 | 26.8% | 62 | 26.8% |  |
| Positive | 2009 | 25.1% | 70 | 29.0% |  | 68 | 29.4% | 68 | 29.4% |  |
| NOS | 3641 | 45.5% | 107 | 44.4% |  | 101 | 43.7% | 101 | 43.7% |  |
| Tumor size (cm) |  |  |  |  | <0.001 |  |  |  |  | 0.616 |
| ≤5cm | 5029 | 62.8% | 133 | 55.2% |  | 124 | 53.7% | 133 | 57.6% |  |
| > 5cm | 2662 | 33.3% | 62 | 25.7% |  | 72 | 31.2% | 62 | 26.8% |  |
| NOS | 312 | 3.9% | 46 | 19.1% |  | 35 | 15.2% | 36 | 15.6% |  |
| MCC: mucinous cell carcinoma; SRCC: signet ring cell carcinoma; RNE: Regional nodes examined; nRT: Neoradiotherapy; RT: Radiotherapy (not neoadjuvant); NOS: Not otherwise specified. | | | | | | | | | | |
